# A YAP–TEAD–CTCF Transcriptional Regulatory Axis Associated With the Elimination of Injured Hepatocytes From the Liver

**DOI:** 10.1111/gtc.70150

**Published:** 2026-10-04

**Authors:** Daiki Hatakeyama, Yoshimi Okamoto‐Uchida, Misaki Kosaka, Haruka Hirose, Satoshi Kofuji, Keiko Danzaki, Yoshiki Endo, Michiko Nakagawa, Akinori Kanai, Yutaka Suzuki, Takeshi Yokoo, Shuji Terai, Teppei Shimamura, Hiroshi Nishina

**Affiliations:** ^1^ Medical Research Laboratory Institute of Integrated Research, Institute of Science Tokyo Tokyo Japan; ^2^ Laboratory of Computational Life Science National Cancer Center Research Institute Tokyo Japan; ^3^ Graduate School of Frontier Sciences The University of Tokyo Chiba Japan; ^4^ Division of Gastroenterology and Hepatology, Graduate School of Medicine, Dentistry and Health Sciences Niigata University Niigata Japan

**Keywords:** CTCF, hepatocyte elimination, spatial transcriptomics, TEAD, YAP

## Abstract

The liver maintains tissue homeostasis both by undergoing regeneration and by eliminating abnormal or injured hepatocytes. The transcriptional coactivator YAP in collaboration with the TEAD family of transcription factors regulates hepatocyte proliferation. It has also been associated with injured hepatocyte elimination whereby YAP‐active injured hepatocytes are extruded into the sinusoidal lumen and undergo cell death followed by macrophage phagocytosis. However, the molecular mechanisms underlying this process are unknown. To address this, we performed RNA‐seq analysis in YAP‐active injured hepatocytes that are eliminated from the liver. We found increased expression of multiple genes associated with cell cycle arrest and cytoskeletal remodeling. Ligand–receptor analysis using single‐cell spatial transcriptomics showed that YAP‐active hepatocytes express the secreted factors CSF1 and CCL2, which promote macrophage recruitment and activation. ATAC‐seq analysis in YAP‐active hepatocytes revealed a significant increase in chromatin accessibility at regions containing CTCF‐binding motifs, and the YAP–TEAD4 complex associated with CTCF in the nucleus. These results indicate that a YAP–TEAD–CTCF axis shapes the gene expression program underlying injured hepatocyte elimination through chromatin structural remodeling, resulting in liver homeostasis.

## Graphical abstract

Injured YAP‐activated hepatocytes are eliminated from the liver. They are extruded toward the sinusoidal lumen, undergo cell death, and are phagocytosed by macrophages. Here, we performed proximity ligation assay, ATAC‐seq, RNA‐seq, and CellChat analyses. Our findings support a model in which the YAP–TEAD complex cooperates with CTCF on chromatin and may promote enhancer–promoter looping to activate a transcriptional program specific to injured hepatocyte elimination.

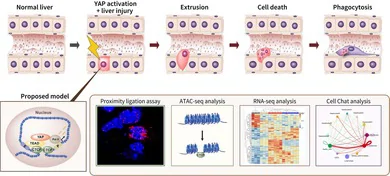

## 1 Introduction

The liver is a central organ for detoxification and metabolism. It is exposed to various stresses, including reactive oxygen species, leading to the emergence of dysfunctional abnormal hepatocytes such as senescent and damaged hepatocytes (Cichoż‐Lach and Michalak 2014; Du et al. 2025; Liu et al. 2020; Ogrodnik et al. 2017). The liver maintains its size and homeostasis through its high regenerative capacity by possessing not only hepatocyte proliferation and repair abilities but also the ability to eliminate abnormal hepatocytes (Liu et al. 2020; Ma et al. 2025). An example of abnormal hepatocyte elimination is the clearance of senescent hepatocytes (Kang et al. 2011). Ras‐induced senescent hepatocytes are eliminated in a CD4^+^ T cell‐dependent manner (Kang et al. 2011; Eggert et al. 2016). In mice lacking adaptive immunity, including T cells and B cells, senescent hepatocytes are not eliminated, leading to the development of liver cancer (Kang et al. 2011).

The transcriptional coactivator YAP regulates liver size through hepatocyte proliferation. When YAP is phosphorylated by the upstream Hippo kinase pathway, it is retained in the cytoplasm and subsequently undergoes protein degradation. Nonphosphorylated YAP translocates into the nucleus, where it binds to TEAD family transcription factors and induces the expression of genes that promote cell proliferation (Camargo et al. 2007; Zhao et al. 2008; Hong and Guan 2012; Zanconato et al. 2016). The constitutively active mutant, YAP (2SA), in which two serine residues phosphorylated by the Hippo pathway are replaced with alanine residues, binds TEAD in the nucleus. We have previously reported that when YAP (2SA) is introduced mosaically into the liver using an adenovirus, hepatocytes proliferate and liver size increases. In contrast, when YAP (2SA) is introduced mosaically into the liver using the hydrodynamic tail vein injection (HTVi), which involves hydrodynamic pressure stimulation, hepatocytes are extruded toward the sinusoidal lumen, undergo cell death, and are phagocytosed by macrophages (Miyamura et al. 2017). Since hepatocyte elimination was also observed in immunodeficient NOG mice, it indicates that adaptive immunity is not essential for this process. Interestingly, when hydrodynamic pressure or ethanol stimulation is applied after introducing YAP (2SA) via an adenovirus, the YAP (2SA)‐expressing hepatocytes are eliminated rather than proliferate. This indicates that injured YAP‐activated hepatocytes are eliminated from the liver. However, the molecular mechanisms involved in this process remain unclear. In particular, it is unclear, Question 1 (Q1) which gene expression programs in injured YAP‐active hepatocytes regulate their elimination, and which expression programs in macrophages respond to those injured hepatocytes by inducing phagocytosis and clearance; Q2 how intercellular interactions between YAP‐active hepatocytes and surrounding cells, including macrophages, are involved in the elimination process; and Q3 if/how chromatin‐level regulatory mechanisms control the expression of genes involved in hepatocyte elimination (Supplementary Figure 1). In this study, we address these issues by leveraging activated YAP (2SA) introduction into mice along with omics analyses.

## 2 Results

### 2.1 Identification of Genes Specifically Associated With YAP‐Dependent Injured Hepatocyte Elimination

The liver is composed of both hepatocytes and nonparenchymal cells, including macrophages. To identify genes essential for YAP‐dependent elimination of injured hepatocytes, we performed comparative RNA‐seq analysis using livers in which activated YAP –YAP (2SA)– was expressed specifically in hepatocytes. This was achieved by delivering YAP (2SA) by AAV (AAV‐YAP liver) to induce hepatocyte proliferation, or by HTVi (HTVi‐YAP liver) to induce hepatocyte elimination (Supplementary Figure 1). As expected, in AAV‐YAP liver, YAP (2SA)‐expressing hepatocytes underwent proliferation, whereas in HTVi‐YAP liver, YAP (2SA)‐expressing hepatocytes underwent elimination (Supplementary Figure 2a,b). In addition, HTVi, but not AAV administration, increased serum levels of ALT, AST, and LDH, confirming acute liver injury under the HTVi condition (Supplementary Figure 2c). We then performed RNA‐seq analysis on RNA extracted from whole livers, followed by hierarchical clustering and heatmap generation (Figure 1a left, whole RNA‐seq, Supplementary Table 1). Samples within the same group showed high similarity, whereas the four groups (no treatment, HTVi‐empty, HTVi‐YAP, and AAV‐YAP) exhibited distinct gene expression profiles (Figure 1b). To identify genes specifically associated with YAP‐dependent elimination of injured hepatocytes, we first identified genes upregulated in each condition (AAV‐YAP vs. no treatment and HTVi‐YAP vs. no treatment; fold change > 2, *p* < 0.05). We then extracted genes that were selectively upregulated in the HTVi‐YAP group but not in the AAV‐YAP group. This analysis identified 1782 genes specific to the HTVi‐YAP group (Figure 1c). Next, YAP‐active hepatocytes were isolated from livers and subjected to the same analysis (Figure 1a right, sort RNA‐seq, Figure 1d,e). This analysis identified 542 genes upregulated in hepatocytes specifically in the HTVi‐YAP group.

**FIGURE 1:**
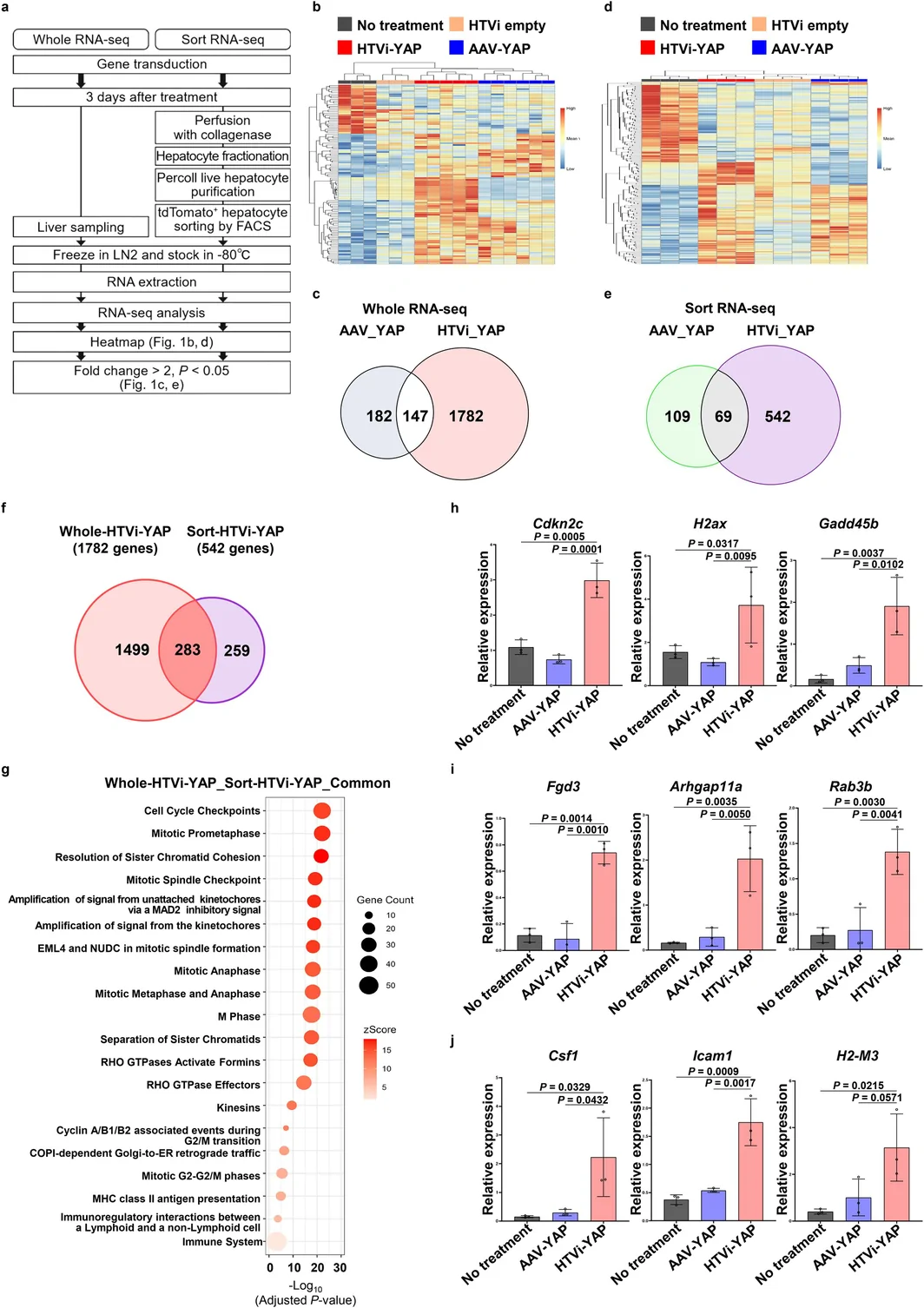
Transcriptome analyses of livers and hepatocytes following YAP induction by AAV or HTVi. (a) Schematic overview of the RNA‐seq workflow for whole liver RNA‐seq (left) and sorted hepatocyte RNA‐seq (right). For sorted RNA‐seq, hepatocytes were isolated by collagenase perfusion and hepatocyte fractionation, followed by purification of live hepatocytes and FACS sorting of tdTomato‐positive hepatocytes. Liver tissues or sorted hepatocytes were collected 3 days after YAP induction and subjected to RNA‐seq analysis. (b) Unsupervised hierarchical clustering heatmap of whole liver RNA‐seq data using differentially expressed genes (DEGs) across the indicated groups (No treatment, HTVi‐empty, HTVi‐YAP, and AAV‐YAP). Red and blue indicate relatively high and low expression, respectively. (c) Venn diagram showing the overlap of significantly upregulated genes (fold change > 2, *p* < 0.05) in the AAV‐YAP and HTVi‐YAP groups compared with the no‐treatment control group in whole liver RNA‐seq. (d) Unsupervised hierarchical clustering heatmap of sorted hepatocyte RNA‐seq data using DEGs across the indicated groups (No treatment, HTVi‐empty, HTVi‐YAP, and AAV‐YAP). Red and blue indicate relatively high and low expression, respectively. (e) Venn diagram showing the overlap of significantly upregulated genes (fold change > 2, *p* < 0.05) in the AAV‐YAP and HTVi‐YAP groups compared with the no‐treatment control group in sorted hepatocyte RNA‐seq. (f) Venn diagram showing the overlap of HTVi‐specifically upregulated genes between whole RNA‐seq analysis and sort RNA‐seq analysis. (g) Reactome pathway analysis of genes commonly upregulated in the HTVi‐YAP group in both whole liver and sorted hepatocyte RNA‐seq datasets. The *x*‐axis indicates −log10 (adjusted *p* value), dot size represents gene count, and color intensity indicates z‐score. (h–j) qPCR validation of representative genes associated with the indicated Reactome pathway terms: Cell cycle checkpoints (h), RHO GTPase effectors (i), and Immunoregulatory interactions between a lymphoid and a nonlymphoid cell (j). Relative mRNA expression was normalized to *Gapdh*. Data are shown as mean ± SD (*n* = 3).

The 1782 genes identified by whole RNA‐seq analysis included genes expressed not only in YAP‐active hepatocytes but also in non‐parenchymal cells. In contrast, the 542 genes identified by sort RNA‐seq analysis enabled specific capture of the gene expression profile of YAP‐active hepatocytes. By comparing these two datasets for commonly upregulated genes, we identified a high‐confidence set of 283 genes that were consistently upregulated in YAP‐active injured hepatocytes (Figure 1f, and see discussion). To identify the biological functions of these 283 hepatocyte elimination–related genes, we performed Reactome pathway analysis. This revealed enrichment of genes involved in cell cycle checkpoints (e.g., *Cdkn2c*, *H2ax*, *Gadd45b*), RHO GTPase effectors (e.g., *Fgd3*, *Arhgap11a*, *Rab3b*), and immunoregulatory interactions between a lymphoid and a nonlymphoid cell (e.g., *Csf1*, *Icam1*, *H2‐M3*) (Figure 1g). *Cdkn2c*, *H2ax*, and *Gadd45b* regulate the G1/S checkpoint, DNA damage checkpoint signaling, and the G2/M checkpoint, respectively (Supplementary Figure 3a). *Fgd3*, *Arhgap11a*, and *Rab3b* regulate RHO GTPase signaling and actin dynamics (Supplementary Figure 3b). *Icam1*, *H2‐M3*, and *Csf1* are involved in immune‐cell adhesion, immune recognition, and macrophage responses, respectively (Supplementary Figure 3c). To validate whether these representative genes included in these terms were specifically upregulated in HTVi‐YAP liver, we performed qPCR using mouse livers (no treatment, HTVi‐YAP, and AAV‐YAP). All of these genes showed specific and significant upregulation in the HTVi‐YAP group (Figure 1h–j). Taken together, these results indicate that genes specifically associated with hepatocyte elimination include those related to negative regulation of the cell cycle, cytoskeletal remodeling, and immune cell recruitment.

### 2.2 Specific Gene Expression Induced in Macrophages Associated With YAP‐Mediated Hepatocyte Elimination

To analyze the different cell populations that constitute the liver from the perspective of gene expression, Visium HD spatial transcriptomic analysis was performed using normal liver (control) and HTVi‐YAP liver at Day 3 after treatment (HTVi‐YAP). For the Control, we utilized a previously reported dataset from our group (Kosaka et al. 2026). For cell segmentation, paraffin‐embedded sections were stained with ATPase Na^+^/K^+^ transporting subunit alpha 1 (ATP1a1) to label cell membranes and DAPI to label nuclei. We obtained over 2.4 billion reads for Control and over 2.9 billion reads for HTVi‐YAP, with sequencing saturation exceeding 87%. After batch correction and data integration, 79,169 cells and 13,462 expressed genes were detected in the Control, and 57,326 cells and 13,469 expressed genes were detected in HTVi‐YAP (Figure 2a).

**FIGURE 2:**
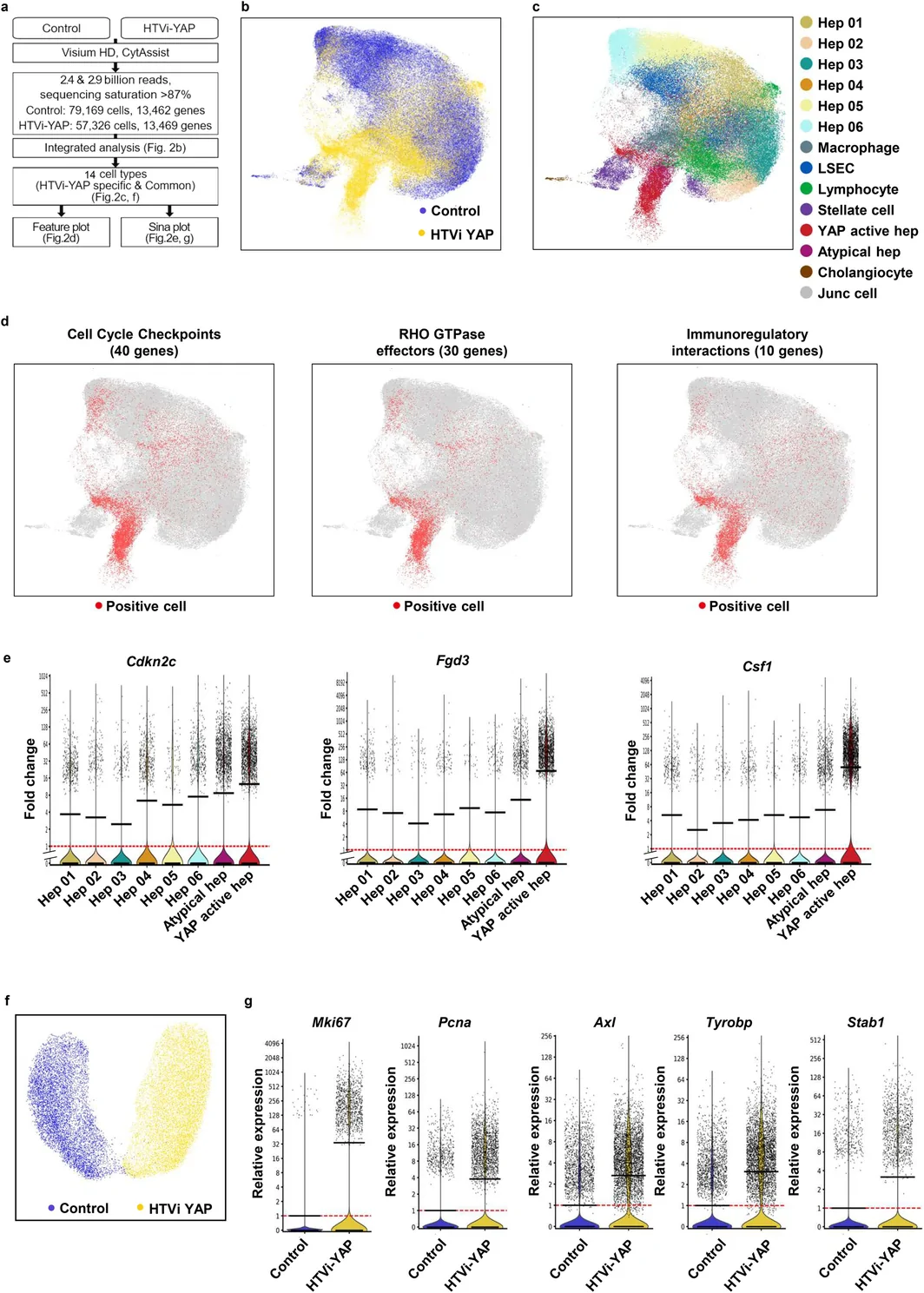
Single‐cell spatial transcriptomic analysis of YAP‐induced liver by Visium HD. (a) Schematic overview of the Visium HD spatial transcriptomic analysis workflow. Liver sections from Control and HTVi‐YAP mice were subjected to Visium HD and analyzed using CytAssist. Integrated analysis identified 14 cell types, including HTVi‐YAP–specific and shared populations. (b) UMAP plot showing the integrated distribution of cells from Control and HTVi‐YAP liver samples. Blue and yellow indicate cells derived from Control and HTVi‐YAP livers, respectively. (c) UMAP plot showing cell‐type annotation based on transcriptomic profiles. (d) UMAP feature plots showing cells enriched for gene signatures associated with HTVi‐specific transcriptional programs: Cell cycle checkpoints (left), RHO GTPase effectors (middle), and Immunoregulatory interactions between a lymphoid and a non‐lymphoid cell (right). Red indicates signature‐positive cells. (e) Sina plots showing the expression levels of representative genes for each functional group: *Cdkn2c* (Cell cycle checkpoints), *Fgd3* (RHO GTPase effectors), and *Csf1* (Immunoregulatory interactions) across different cell types. Each dot represents the expression value in an individual cell, with the horizontal bar indicating the mean value. Values are normalized relative to the mean expression in hepatocytes from the Control liver (defined as 1). The *Y*‐axis is presented on a linear scale from 0 to 1 and a log_2_ scale for values above 1. (f) UMAP plot showing the distribution of macrophages derived from Control and HTVi‐YAP livers. Blue and yellow indicate macrophages from Control and HTVi‐YAP livers, respectively. (g) Sina plots comparing the expression of proliferation‐associated genes (*Mki67* and *Pcna*) and phagocytosis‐associated genes (*Axl*, *Tyrobp*, and *Stab1*) in macrophages from Control and HTVi‐YAP livers. Each dot represents one cell, and horizontal bars indicate the mean value. Expression levels were normalized to the mean expression in macrophages from Control livers (defined as 1). The *y*‐axis is shown on a linear scale from 0 to 1 and a log_2_ scale for values above 1.

Dimensionality reduction using Uniform Manifold Approximation and Projection (UMAP) revealed both shared and HTVi‐YAP–specific cell populations (Figure 2b). Annotation based on known marker genes identified 14 cell types, including YAP‐active hepatocytes, six hepatocyte subtypes (Hep 01–06), and macrophages (Figure 2c, Supplementary Figure 4). YAP‐active hepatocytes accounted for 23.2% of the total hepatocytes in HTVi‐YAP liver.

To assess whether the 283 hepatocyte elimination–associated genes we identified (Figure 1f) are expressed specifically in the YAP‐active hepatocytes, we visualized the gene sets identified in Figure 1g (cell cycle checkpoints, RHO GTPase effectors, and immunoregulatory interactions between a lymphoid and a nonlymphoid cell) on UMAP (Figure 2d). Cells strongly expressing these gene sets overlapped with YAP‐active hepatocytes. Expression of representative genes within these pathways was further compared across hepatocyte populations (YAP‐active hepatocytes, Hep 01–06, and Atypical hepatocytes) (Figure 2e), revealing significant upregulation of *Cdkn2c*, *Fgd3*, and *Csf1* in YAP‐active hepatocytes.

To examine whether YAP‐mediated hepatocyte elimination induces transcriptional changes also in macrophages responsible for phagocytosis, we compared gene expression profiles in macrophages from Control and HTVi‐YAP livers (Figure 2f). Macrophages from HTVi‐YAP exhibited markedly distinct gene expression profiles compared with Control macrophages. Specifically, proliferation markers (*Mki67*, *Pcna*) and phagocytosis‐related markers (*Axl*, *Tyrobp*, *Stab1*) were significantly upregulated in HTVi‐YAP‐derived macrophages (Figure 2g), indicating macrophage activation and enhanced phagocytic activity. Thus, YAP activation affects gene expression not only in hepatocytes but also in surrounding macrophages.

### 2.3 YAP‐Active Hepatocytes Interact With Macrophages Through Chemokines and Cytokines

To determine how YAP‐active hepatocytes and macrophages communicate and through which molecules, we performed ligand–receptor analysis using RNA‐seq and Visium HD data. From the 283 hepatocyte elimination–associated genes (Figure 1f), we extracted genes encoding extracellular factors. Thirty ligands that are either secreted or presented on the cell surface were specifically upregulated in YAP‐active injured hepatocytes (Figure 3a). To quantify the upregulation of these ligands in YAP‐active injured hepatocytes and the upregulation of their receptors in the whole liver, we performed communication analysis using whole RNA‐seq and sort RNA‐seq data (Figure 3b). The top‐ranked ligand–receptor pairs included chemokine–chemokine receptor pairs involved in the recruitment of macrophages and other immune cells to tissues, such as Ccl2‐Ccr2, Ccl7‐Ccr1/2/3, and Cxcl16‐Cxcr6, as well as the cytokine–cytokine receptor pair Csf1‐Csf1r, which is involved in macrophage proliferation, survival, and phagocytosis.

**FIGURE 3:**
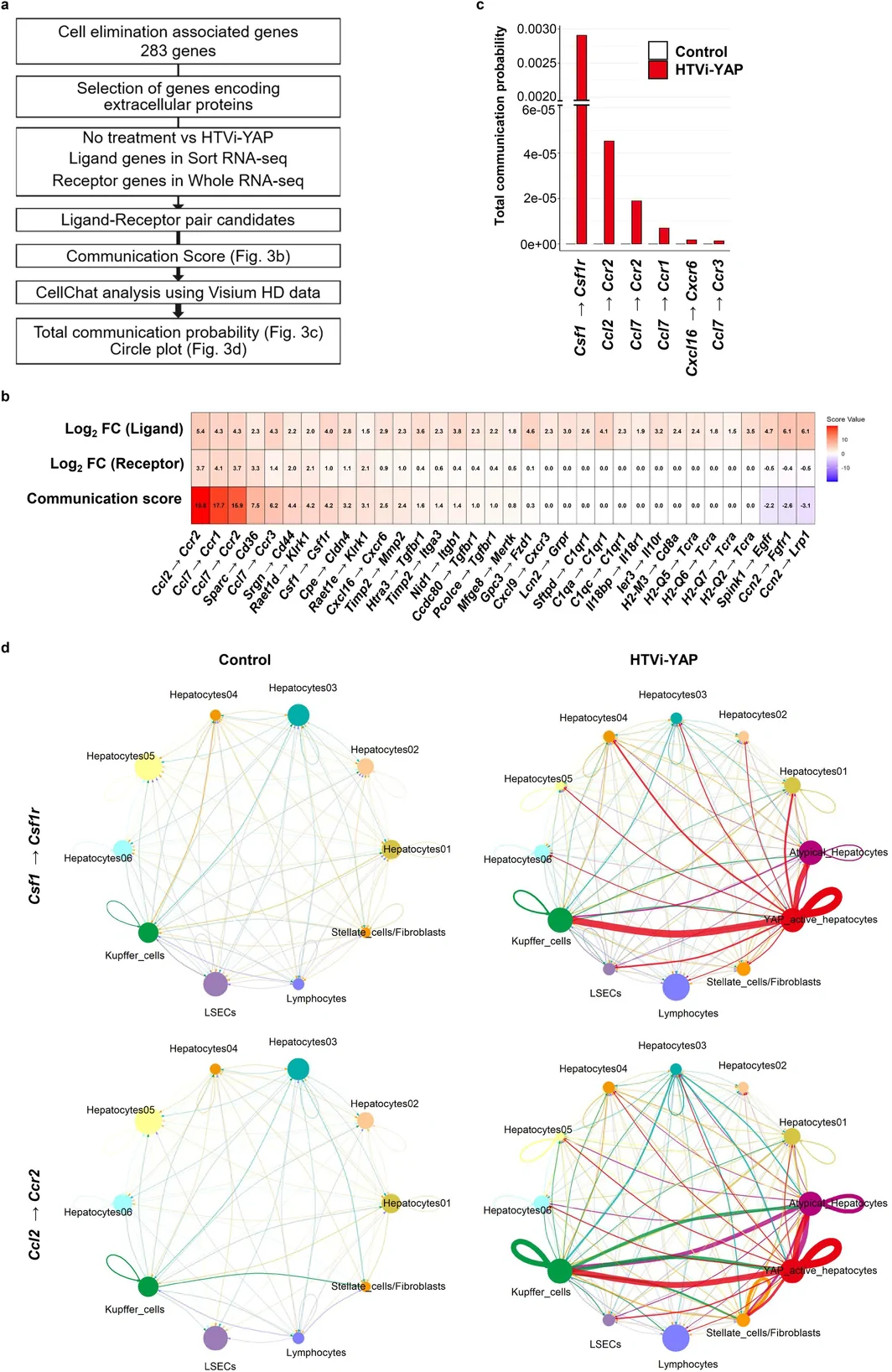
Ligand–receptor interaction analysis between YAP‐active hepatocytes and surrounding cells. (a) Schematic overview of the ligand–receptor analysis workflow. Genes associated with HTVi‐specific transcriptional changes (283 genes) were filtered for those encoding extracellular proteins. Candidate ligand–receptor pairs were then identified by combining ligand genes upregulated in sorted HTVi‐YAP hepatocytes (sort RNA‐seq) with receptor genes upregulated in whole HTVi‐YAP livers (whole RNA‐seq), followed by prioritization using a communication score and CellChat analysis of Visium HD data. (b) Heatmap showing ranked ligand–receptor pair candidates based on the communication score. The communication score was calculated as the product of the log_2_ fold change of ligand expression in sorted HTVi‐YAP hepatocytes and the log_2_ fold change of receptor expression in whole HTVi‐YAP livers. The log_2_ fold change values for each ligand and receptor, as well as the resulting communication score, are shown. Red indicates higher values. (c) Comparison of the total communication probability inferred from Visium HD data between Control and HTVi‐YAP groups for the top‐ranked ligand–receptor pairs identified by the communication score. (d) Circle plots showing cell‐type–to–cell‐type communication probabilities for representative ligand–receptor pairs. Upper panels, Csf1–Csf1r interaction in Control (left) and HTVi‐YAP (right) groups. Lower panels, Ccl2–Ccr2 interaction in Control (left) and HTVi‐YAP (right) groups. Edge thickness indicates the inferred communication probability between the indicated cell types.

To confirm ligand–receptor interactions between YAP‐active hepatocytes and macrophages or surrounding cells in the HTVi‐YAP liver, we performed CellChat analysis using Visium HD data for Control and HTVi‐YAP (Figure 3a) (Jin et al. 2021). Six ligand–receptor pairs showing significant communication in HTVi‐YAP were detected (Figure 3c). To determine which cell types were involved in these interactions, we generated circle plots for the strongly involved ligand–receptor pairs *Csf1‐Csf1r* and *Ccl2‐Ccr2*. In Control liver, the signal strength of *Csf1‐Csf1r* between hepatocytes and macrophages was extremely weak, and no prominent interactions were observed among other cell types (Figure 3d upper‐left). In HTVi‐YAP liver, YAP‐active hepatocytes functioned as the major signal senders by expressing *Csf1*, whereas macrophages functioned as receivers by expressing *Csf1r* (Figure 3d upper‐right). Similarly, *Ccl2‐Ccr2* appeared to function through secretion of *Ccl2* by YAP‐active hepatocytes and expression of *Ccr2* by macrophages (Figure 3d lower‐right). These results indicate that during YAP‐mediated hepatocyte elimination, YAP‐active hepatocytes specifically interact with macrophages through chemokines and cytokines such as *Csf1* and *Ccl2*.

### 2.4 In YAP‐Activated Injured Hepatocytes, CTCF‐Binding Sites Are Specifically Converted Into an Open Chromatin State

To identify the regulatory mechanisms underlying the gene expression programs associated with hepatocyte elimination, we performed ATAC‐seq analysis. First, to identify injury‐dependent open chromatin regions, hepatocytes were isolated from livers in which YAP induced hepatocyte proliferation and from livers in which YAP induced hepatocyte elimination, followed by ATAC‐seq analysis (Figure 4a). To determine which factors bind to chromatin regions specifically open in HTVi‐YAP hepatocytes, we performed motif analysis. CTCF‐binding motifs were found to be the most significantly enriched (Figure 4b). CTCF‐bound genomic regions are often identified as open chromatin. Upon binding to chromatin, CTCF cooperates with factors such as cohesin to form DNA loops, thereby regulating gene expression in both activating and repressive contexts (Ong and Corces 2014; de Wit et al. 2015; Sun et al. 2025). In addition, binding motifs of multiple nuclear factors, including COUP TFII, were significantly enriched.

**FIGURE 4:**
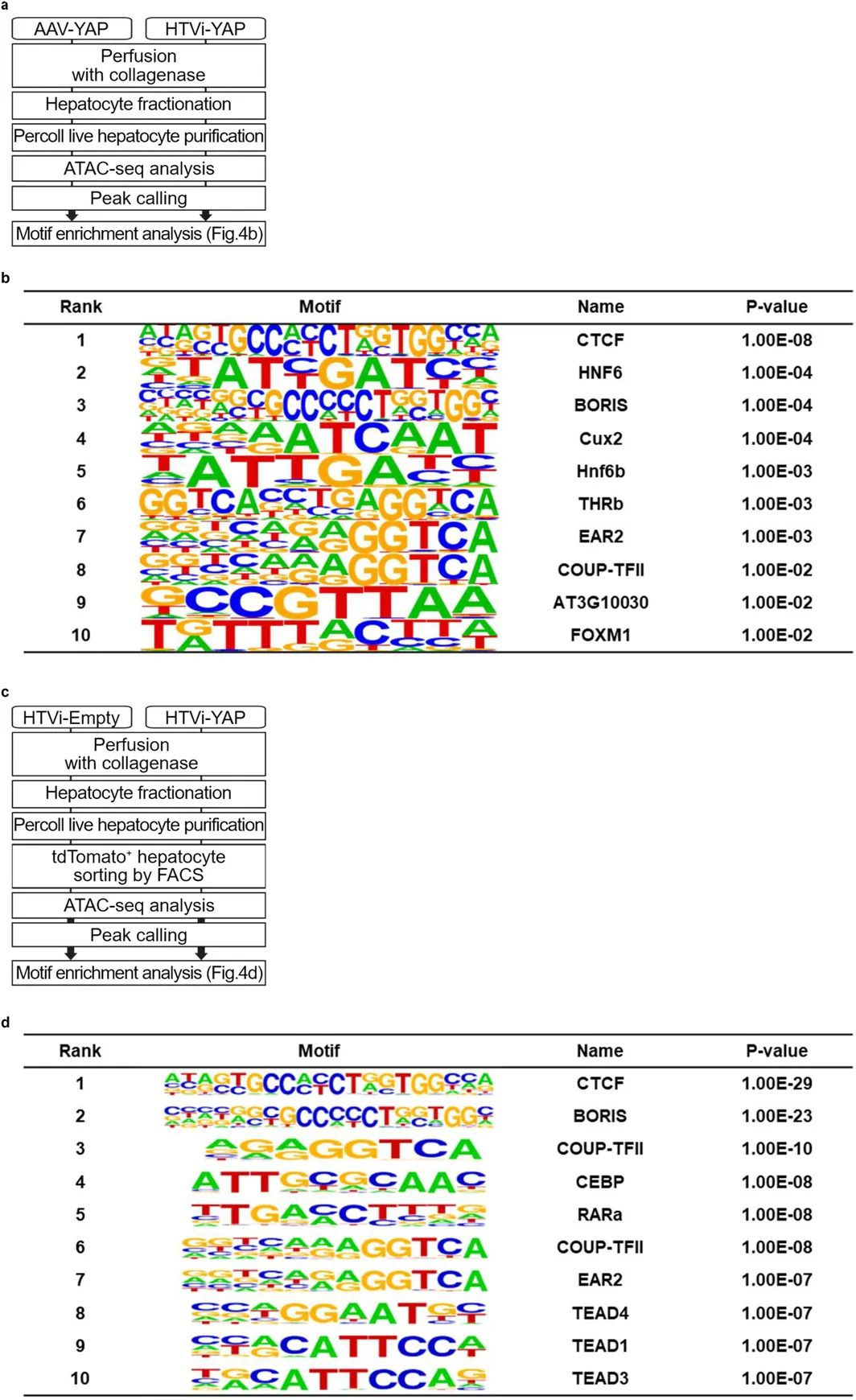
Identification of open chromatin regions specifically induced in YAP‐active injured hepatocytes. (a) Schematic overview of the comparative ATAC‐seq analysis workflow between hepatocytes isolated from AAV‐YAP and HTVi‐YAP livers. (b) Transcription factor binding motifs enriched in HTVi‐YAP–specific open chromatin regions compared with AAV‐YAP livers. The top 10 motifs ranked by statistical significance are shown. (c) Schematic overview of the comparative ATAC‐seq analysis workflow between tdTomato‐positive hepatocytes isolated from HTVi‐Empty and HTVi‐YAP livers. (d) Transcription factor binding motifs enriched in HTVi‐YAP–specific open chromatin regions compared with HTVi‐Empty livers. The Top 10 motifs ranked by statistical significance are shown.

Next, to identify specifically YAP‐dependent open chromatin regions, hepatocytes expressing control vector or YAP (2SA) were isolated from HTVi‐Empty or HTVi‐YAP livers and subjected to ATAC‐seq analysis (Figure 4c). Motif analysis revealed that CTCF motifs were again the most significantly enriched (Figure 4d). In addition, significant enrichment of binding motifs for TEAD1/3/4, which are major transcriptional partners of YAP, as well as COUP TFII and CEBP, was observed. Taken together, these results suggest that CTCF‐mediated DNA looping occurs at open chromatin regions in a YAP‐ and injury‐dependent manner.

### 2.5 YAP Interacts With CTCF Via TEAD

CTCF was recently shown to cooperate with the YAP‐TEAD complex by interacting with YAP in human ES cells in response to mechanical cues to regulate gene expression (Sun et al. 2025). To examine whether the YAP–TEAD complex interacts with CTCF in hepatocytes, we performed structural prediction by using AlphaFold3. AlphaFold3 enables prediction of three‐dimensional protein structures from primary amino acid sequences and allows identification of potential interaction interfaces (Abramson et al. 2024; Jumper et al. 2021). As expected, AlphaFold3 predicted strong interaction signals between the N‐terminal TEAD‐binding domain of YAP and the YAP‐binding domain of TEAD4 (Figure 5a, upper‐left, Figure 5b) (Vassilev et al. 2001; Li et al. 2010; Chen et al. 2010). Next, we examined a possible interaction between TEAD and CTCF. Interestingly, signals indicating structural stability were detected between the TEA domain and YAP‐binding domain of TEAD1/2/3/4 and the zinc finger domain of CTCF (Figure 5a, middle and right columns, Figure 5b). In contrast, no signals indicating structural stability were observed between YAP and CTCF (Figure 5a, lower‐left). These results suggest that TEAD, but not YAP, interacts with CTCF.

**FIGURE 5:**
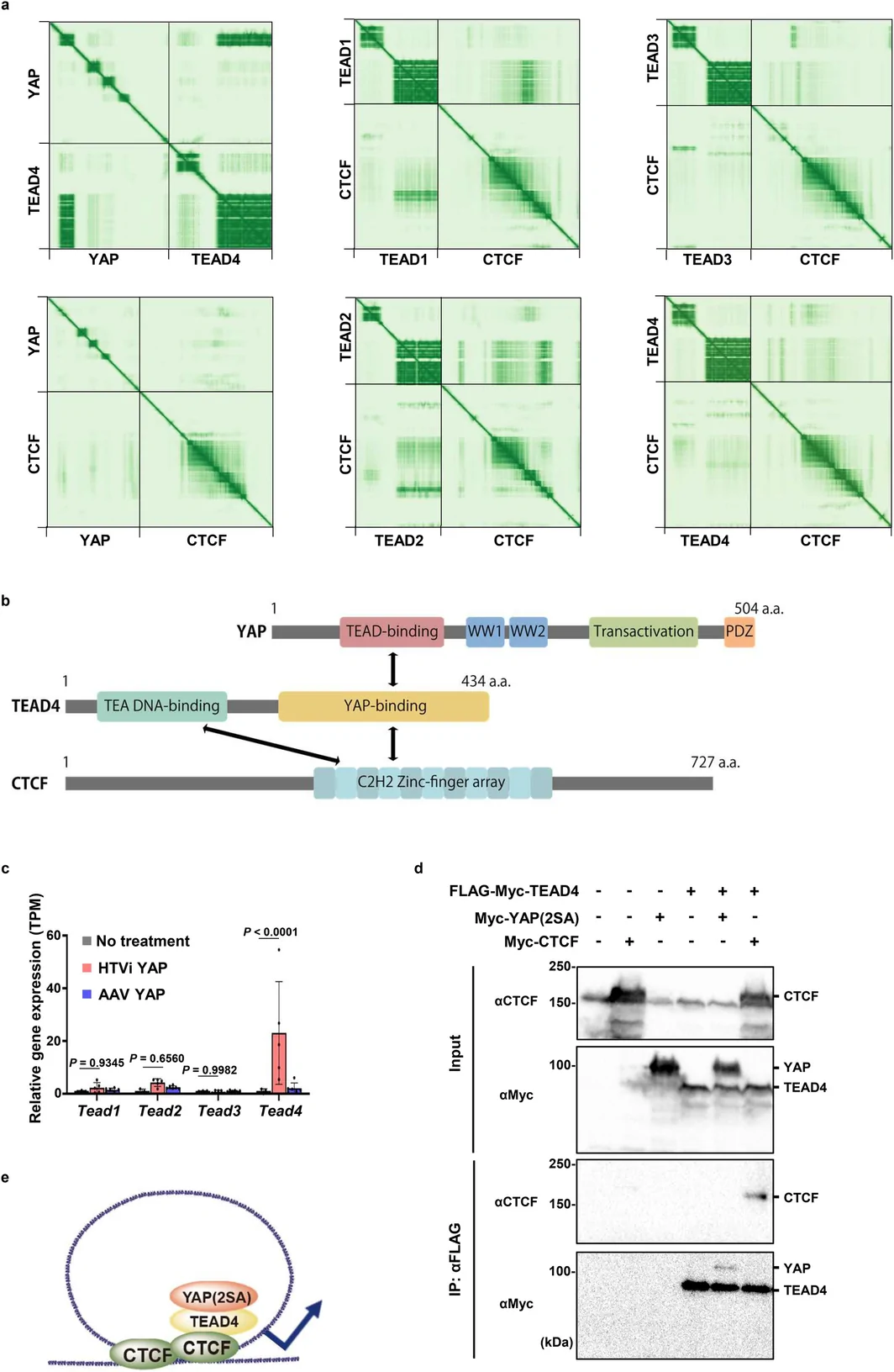
Analysis of the interaction between YAP–TEAD and CTCF. (a) Predicted Aligned Error (PAE) plots for AlphaFold 3 structural predictions of the YAP–TEAD4, YAP–CTCF, and TEAD family–CTCF complexes. Darker green indicates lower predicted aligned error and thus higher confidence in the predicted relative positioning of residues. (b) Schematic representation of the domain structures of YAP, TEAD4, and CTCF. Arrows indicate the known YAP–TEAD4 interaction and the TEAD4–CTCF interaction regions predicted by AlphaFold 3. (c) Relative expression levels of *Tead* family genes in each group (No treatment, AAV‐YAP, and HTVi‐YAP) obtained from whole liver RNA‐seq. Data are shown as mean ± SD. Statistical significance was assessed by one‐way ANOVA. (d) Co‐immunoprecipitation analysis of TEAD4 and CTCF interaction in HEK293T cells. Representative immunoblot showing that Myc‐CTCF was co‐immunoprecipitated with FLAG‐TEAD4. Myc‐YAP(2SA) was included as a positive control for TEAD4 binding. (e) Proposed model in which the YAP(2SA)–TEAD4 complex cooperates with CTCF to activate transcription of target genes. CTCF‐mediated chromatin architecture may facilitate recruitment or stabilization of the YAP–TEAD4 transcriptional complex at regulatory elements associated with hepatocyte elimination.

Among the four TEAD family members, *Tead4* gene expression was specifically increased in HTVi‐YAP liver (Figure 5c); therefore, subsequent analyses focused on TEAD4. To examine a potential interaction between TEAD4 and CTCF in cells, we performed co‐immunoprecipitation experiments. In addition to the band corresponding to the YAP–TEAD4 interaction, a band indicating interaction between TEAD4 and CTCF was detected (Figure 5d). These results suggest that the YAP/TEAD4 complex interacts with CTCF via TEAD4 (Figure 5e).

### 2.6 YAP–TEAD and CTCF Form a Nuclear Ternary Complex

To determine whether YAP–TEAD complexes associate with CTCF in the nucleus, which would indicate they form a functional complex to regulate transcription, we performed a proximity ligation assay (PLA) using the human hepatocellular carcinoma cell line, HepG2. PLA enables in situ visualization of protein proximity. Using anti‐YAP and anti‐CTCF antibodies, we assessed the spatial proximity between YAP and CTCF (Figure 6a). In untreated control HepG2 cells (in the absence of YAP (2SA)), no PLA signal indicative of YAP–CTCF proximity was detected (Figure 6b, upper). In contrast, HepG2 cells expressing activated YAP (in the presence of YAP (2SA)) showed focal PLA signals within the nucleus (Figure 6b, lower). Taken together, our results support a model in which a YAP–TEAD–CTCF axis activates a gene expression program that mediates the elimination of injured hepatocytes from the liver.

**FIGURE 6:**
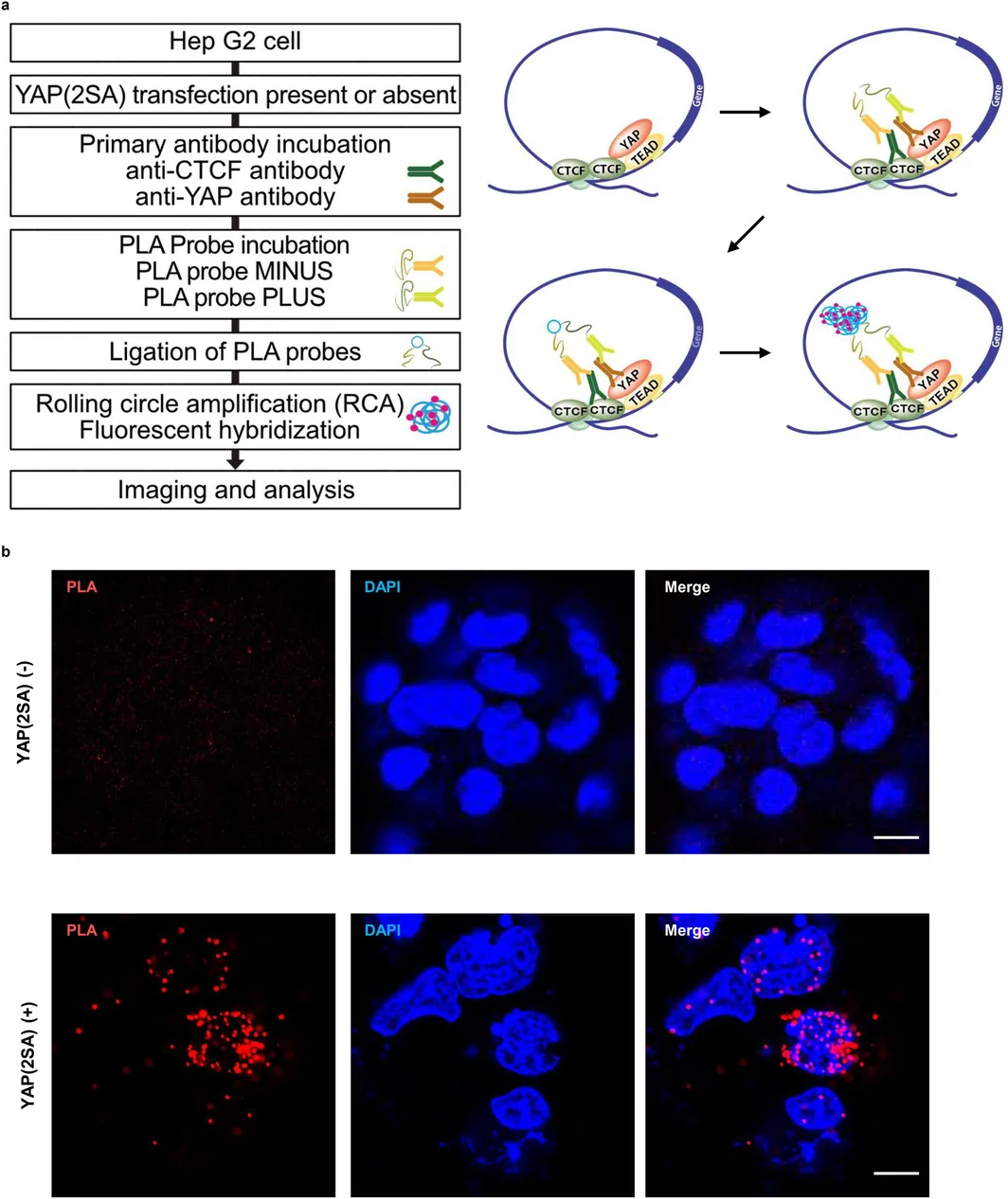
Proximity ligation assay for YAP and CTCF. (a) Schematic overview of the in situ PLA workflow and detection principle. (b) Representative images of in situ PLA for YAP and CTCF in HepG2 cells with or without YAP(2SA) transfection. Red indicates PLA signals and blue indicates DAPI. Scale bar, 10 μm.

## 3 Discussion

In this study, integration of whole RNA‐seq and sort RNA‐seq datasets enabled the identification of 283 genes reproducibly associated with YAP‐dependent elimination of injured hepatocytes (Figure 1). Among the 542 genes identified by sort RNA‐seq, 283 overlapped with whole RNA‐seq, whereas the remaining 259 genes were unique to sort RNA‐seq. A key methodological difference between these approaches is the sampling procedure. In whole RNA‐seq, liver tissue was frozen within 5 min of dissection, whereas in sort RNA‐seq, collagenase digestion and FACS isolation of YAP‐active hepatocytes required 2–3 h. It is therefore likely that some of the 259 genes identified by sort RNA‐seq but not detected in whole RNA‐seq were induced during tissue dissociation and cell isolation, including so‐called dissociation‐induced genes (van den Brink et al. 2017; Denisenko et al. 2020). By excluding sort RNA‐seq–specific genes, we were able to minimize artifacts introduced during cell isolation and define a high‐confidence set of 283 genes consistently upregulated in YAP‐active injured hepatocytes.

CSF1 and CCL2 were identified as signals mediating the interaction between YAP‐active injured hepatocytes and macrophages (Figure 3b). CCL2 recruits CCR2‐positive inflammatory monocytes from outside the liver into hepatic tissue (Liao et al. 2023). Thus, the macrophages responsible for cell elimination in this context may include not only Kupffer cells but also monocyte‐derived macrophages. CSF1 is well known to promote macrophage survival, proliferation, and phagocytic activity (Keshvari et al. 2021; Sehgal et al. 2021). In our experimental system, YAP‐active injured hepatocytes protrude into the sinusoidal lumen, undergo cell death, and are subsequently engulfed by macrophages (Miyamura et al. 2017). We found that macrophages exhibited increased expression of *Stab1*, *Tyrobp*, and *Axl* (Figure 2g). Stab1 is induced by CSF1 stimulation and functions as a receptor that directly recognizes phosphatidylserine (PS), an eat‐me signal exposed on dying cells, thereby mediating their engulfment (Brochériou et al. 2011). *Tyrobp* is also induced in response to CSF1 stimulation and acts as an adaptor molecule that associates with CSF1R and phagocytic receptors such as TREM2, thereby amplifying macrophage activation and phagocytosis via Syk kinase signaling (Otero et al. 2009). Axl functions as a phagocytic receptor by recognizing Gas6‐bound PS on the surface of dying cells. Taken together, these findings indicate that CSF1 secreted by YAP‐active hepatocytes enhances the expression of phagocytosis‐related receptors in macrophages, thereby promoting their own engulfment and elimination.

Our ATAC‐seq analysis revealed that regions showing increased chromatin accessibility in response to both YAP activation and injury were enriched not only for CTCF‐ and TEAD‐binding motifs, but also for motifs recognized by transcription factors such as COUP‐TFII and C/EBP (Figure 4b,d). COUP‐TFII positively regulates *CCL2* gene expression in astrocytes, while C/EBP is required for insulin‐induced *CCL2* gene expression in vascular smooth muscle cells (Delépine et al. 2015; Sato et al. 2007). In addition, both COUP‐TFII and C/EBP have been reported to cooperate with or interact with the YAP‐TEAD complex (Abraham et al. 2025; Kohli et al. 2014). Furthermore, structural prediction using AlphaFold3 also suggested that COUP‐TFII and C/EBP can each interact with TEAD4 (Supplementary Figure 5). Taken together, these findings indicate that, in YAP‐active injured hepatocytes, YAP/TEAD cooperates not only with CTCF but also with multiple transcription factors such as COUP‐TFII and C/EBP to regulate the expression of genes associated with injured hepatocyte elimination.

The YAP/TEAD complex binds predominantly to distal enhancer regions rather than promoters to regulate the expression of target genes (Zanconato et al. 2015; Biagioni et al. 2022). In this context, Sun et al. recently reported that, in human ES cells, the YAP‐TEAD complex cooperates with CTCF in response to extracellular mechanical cues to regulate gene expression through enhancer–promoter (E–P) interactions (Sun et al. 2025). They also proposed that CTCF interacts with YAP. Our results are broadly consistent with the model proposed by Sun et al., while further suggesting that the association between YAP‐TEAD and CTCF may be mediated by TEAD in the nucleus (Figures 5 and 6). The system used by Sun et al. might have included endogenous TEAD. Taken together, these findings support a model whereby the YAP‐TEAD–CTCF axis mediates injured hepatocyte elimination through distal enhancers and E–P looping (Figure 7).

**FIGURE 7:**
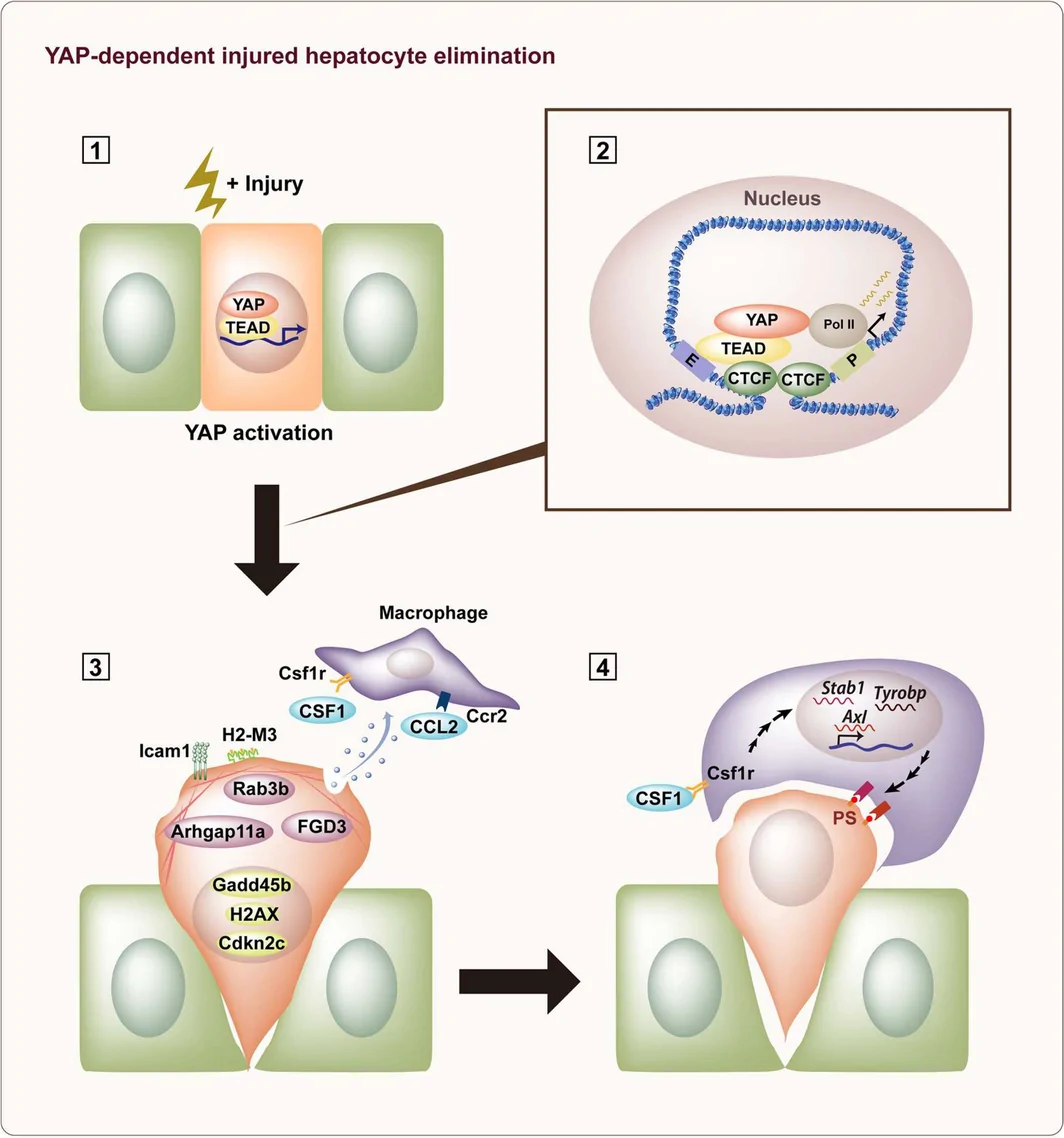
Proposed model for injured YAP‐active hepatocyte elimination. (1) Injury stimuli are imposed on hepatocytes with activated YAP signaling. (2) The YAP–TEAD complex cooperates with CTCF on chromatin and may promote enhancer–promoter looping to activate a distinct transcriptional program. (3) In YAP‐active hepatocytes, genes associated with cell cycle checkpoints (e.g., *Cdkn2c*, *H2ax*, and *Gadd45b*), RHO GTPase effectors (e.g., *Fgd3*, *Arhgap11a*, and *Rab3b*), and immunoregulatory interactions (e.g., *Csf1*, *Ccl2*, *Icam1*, and *H2‐M3*) are induced. (4) Signals such as CSF1 promote macrophage activation, leading to induction of phagocytosis‐associated genes (e.g., *Stab1*, *Tyrobp*, and *Axl*) and elimination of YAP‐active hepatocytes. E, enhancer; P, promoter; Pol II, RNA polymerase II; PS, phosphatidylserine.

Another example of abnormal hepatocyte elimination, oncogenic RAS‐dependent clearance of senescent hepatocytes, has been reported (Kang et al. 2011; Eggert et al. 2016; Sturmlechner et al. 2021). Hepatocytes expressing RAS (G12V) secrete chemokines, such as CCL2 and CXCL14, which induce macrophage activation and T cell responses, thereby promoting cell elimination. In contrast, YAP‐activated injured hepatocytes have been reported to be phagocytosed by macrophages, likely in response to apoptosis‐induced “eat‐me” signals (Miyamura et al. 2017). In this study, we found that YAP‐activated injured hepatocytes secrete CCL2 and CSF1 (Figures 2, 3, and 7). These findings suggest that CCL2 and CSF1 contribute to macrophage recruitment and phagocytosis, although their requirement for this process remains to be directly tested.

We have previously found that elimination of YAP‐active hepatocytes is induced not only by the rapid hydrodynamic stimulus caused by HTVi, but also by ethanol administration (Miyamura et al. 2017). Thus, physical changes mediated through the cell membrane may serve as common upstream signals (Wondergem and Davis 1994; Vom Dahl and Häussinger 1998). However, the precise signaling mechanisms remain unclear. Although our ATAC‐seq and motif analyses suggest that CTCF motif‐containing open chromatin regions are associated with HTVi‐YAP‐specific gene expression, this study did not directly demonstrate CTCF binding or CTCF‐mediated chromatin looping at the genomic regions of individual hepatocyte elimination‐associated genes. Further analyses, including CTCF ChIP‐seq and Hi‐C, will be required to clarify these genome‐structural mechanisms. In addition, it remains incompletely understood how much the clearance of abnormal cells, such as senescent and injured hepatocytes, contributes to tissue homeostasis in vivo. These issues represent an important area for future investigation.

## 4 Experimental Procedures

### 4.1 Mice

C57BL/6J mice were purchased from Sankyo Labo Service Corporation (Tokyo, Japan). tdTomato reporter mice [B6.Cg‐Gt(ROSA)26Sor(CAG‐tdTomato)Hze/J] were purchased from The Jackson Laboratory (Bar Harbor, ME, USA). Eight‐week‐old male mice were used in this study. Mice were housed under specific pathogen‐free conditions on a 12 h light/dark cycle with free access to water and a radiation‐sterilized CE‐2 diet (CLEA Japan, Tokyo, Japan). All animal experiments were approved by the Institutional Animal Care and Use Committee of the Institute of Science Tokyo (Approval No. A2025‐019A). All experiments were performed in accordance with institutional guidelines.

### 4.2 Hydrodynamic Tail Vein Injection

The pLive‐FLAG‐Myc‐YAP (2SA) vector was designed to express FLAG‐ and Myc‐tagged YAP (2SA), a constitutively active form of YAP, under the control of the liver‐specific albumin (Alb) promoter. Additionally, pLive‐FLAG‐Myc‐YAP (2SA)‐IRES‐Cre was generated to co‐express constitutively active YAP and Cre recombinase. The pLive‐empty vector and pLive‐IRES‐Cre vector were used as controls. Hydrodynamic tail vein injection (HTVi) was performed to deliver plasmid DNA into the mouse liver. A total of 20 μg of plasmid DNA was diluted in 2.0 mL of TransIT‐EE Hydrodynamic Delivery Solution (Mirus Bio, Madison, Wisconsin, USA). The DNA solution was rapidly injected into the tail vein within 5 s (Miyamura et al. 2017; Liu et al. 1999).

### 4.3 Adeno‐Associated Virus Administration

Two adeno‐associated virus serotype 8 (AAV8) vectors were used in this study. AAV8‐Alb‐FLAG‐Myc‐YAP(2SA) was used to express FLAG‐ and Myc‐tagged YAP(2SA) under the albumin promoter, and AAV8‐TBG‐FLAG‐Myc‐YAP(2SA)‐IRES‐Cre was used to co‐express YAP(2SA) and Cre recombinase. Both vectors were custom‐produced by VectorBuilder Inc. (Chicago, Illinois, USA). Mice were injected via the tail vein with 3.0 × 10^11^ genome copies (gc) of AAV diluted in 300 μL PBS.

### 4.4 Measurement of Serum AST, ALT, and LDH Levels

Blood was collected by terminal orbital enucleation. Serum was isolated by centrifugation at 1000 × g for 10 min at 4°C, transferred to a new tube, and centrifuged again under the same conditions to remove residual cellular debris. Serum AST, ALT, and LDH levels were measured using a SPOTCHEM EZ automated analyzer with SPOTCHEM II Liver‐1 reagent strips (ARKRAY, Kyoto, Japan) according to the manufacturer's instructions.

### 4.5 Tissue Preparation for Histological Analysis

Liver tissues were harvested and fixed in 10% neutral buffered formalin (Nacalai Tesque, Kyoto, Japan) for 24 h. For paraffin embedding, fixed tissues were processed using an Excelsior ES tissue processor and embedded using a HistoStar embedding center (Thermo Fisher Scientific, Waltham, Massachusetts, USA). Paraffin blocks were sectioned at 5 μm. For frozen sectioning, fixed tissues were washed with PBS, cryoprotected overnight in 30% sucrose in PBS at 4°C, embedded in OCT compound (Sakura Finetek, Tokyo, Japan), and stored at −80°C until sectioning. Frozen blocks were sectioned at 8 μm and air‐dried for 3 h before use.

### 4.6 Immunofluorescence Staining

Sections were washed with PBS and blocked/permeabilized for 30 min at RT in blocking buffer containing 5% BSA, 0.1% Triton X‐100, and 0.03% sodium azide in TBS. Sections were incubated overnight at 4°C with anti‐c‐Myc antibody (1:500; #C3956; Sigma‐Aldrich, St. Louis, Missouri, USA) diluted in blocking buffer. After washing with PBS containing 0.1% Triton X‐100, sections were incubated for 1 h at RT with Alexa Fluor 647‐conjugated anti‐rabbit IgG secondary antibody (1:500; #4414S; Cell Signaling Technology) and DAPI (1:1000). Sections were then washed and mounted with Mowiol mounting medium.

### 4.7 Image Acquisition and Quantification

Images were acquired using a BZ‐X700 fluorescence microscope (Keyence, Osaka, Japan) and analyzed with BZ‐X Analyzer software (Keyence). For quantification of YAP‐positive hepatocytes, hepatocytes were identified based on nuclear area using a threshold set at approximately 35 μm^2^, which was applied consistently across all samples in each experiment. The percentage of YAP‐positive hepatocytes was calculated as the number of Myc‐positive cells divided by the total number of identified hepatocytes.

### 4.8 Quantitative Real‐Time PCR

Total RNA was extracted using TRIzol Reagent (Invitrogen, Carlsbad, California, USA). cDNA was synthesized using ReverTra Ace qPCR RT Master Mix (Toyobo, Osaka, Japan). Quantitative real‐time PCR was performed using THUNDERBIRD SYBR qPCR Mix (Toyobo) on a CFX96 Real‐Time PCR System (Bio‐Rad, Hercules, California, USA). Cycling conditions were 95°C for 30 s, followed by 40 cycles of 95°C for 5 s and 60°C for 30 s. Primer sequences are listed in Supplementary Table 2.

### 4.9 Hepatocyte Isolation and Flow Cytometric Sorting

Mice were anesthetized by intraperitoneal injection of a triple anesthetic mixture (10 μL/g body weight), consisting of midazolam (4 mg/kg), medetomidine (0.75 mg/kg), and butorphanol (5 mg/kg). Livers were perfused in situ through the portal vein using a 24‐gauge catheter. The liver was first perfused with 20 mL of Liver Perfusion Medium prewarmed to 37°C at 5 mL/min, followed by 20 mL of Liver Digest Medium containing collagenase I at the same flow rate. After digestion, livers were excised, the gallbladder was removed, and tissues were mechanically dissociated in DMEM. Cell suspensions were filtered through a 70 μm cell strainer and centrifuged at 50 × g for 1 min at 4°C. The pellet was resuspended in PBS and mixed 1:1 with isotonic Percoll solution (9 parts Percoll and 1 part 10× PBS), followed by centrifugation at 50×*g* for 10 min at 4°C. Viable hepatocytes were washed twice with PBS (100×*g*, 1 min, 4°C), resuspended in hepatocyte culture medium, filtered through a 40 μm cell strainer, and stained with DAPI. Flow cytometric sorting was performed using a FACSAria III cell sorter (BD Biosciences, San Jose, California, USA). Live hepatocytes were defined as DAPI‐negative cells, and tdTomato‐positive hepatocytes were collected.

### 4.10 RNA‐Seq

Total RNA was extracted using the RNeasy Kit (QIAGEN, Hilden, Germany) according to the manufacturer's instructions. RNA sequencing was performed by Takara Bio Inc. (Shiga, Japan). cDNA amplification and library preparation were performed using the SMART‐seq v4 Ultra Low Input RNA Kit for Sequencing (Clontech, Mountain View, California, USA), Nextera XT DNA Library Prep Kit, and Nextera XT Index Kit v2 (Illumina, San Diego, California, USA). Libraries were sequenced on a NovaSeq 6000 platform. Sequence data were processed using DRAGEN Bio‐IT Platform v3.6.3 (Illumina) with GRCm38 release m25 as the reference genome. Differential gene expression analysis was performed based on transcripts per million (TPM) values.

### 4.11 Communication Score Analysis

To estimate ligand–receptor‐based intercellular communication initiated by YAP‐active hepatocytes, a communication score was defined using sorted hepatocyte RNA‐seq and whole‐liver RNA‐seq datasets. Ligands were selected as genes encoding secreted proteins that were significantly upregulated in the HTVi‐YAP group relative to the no‐treatment group in the sorted hepatocyte RNA‐seq dataset (fold change ≥ 2 and *p* < 0.05). Cognate receptors were identified based on the literature, and receptor expression was evaluated in the whole‐liver RNA‐seq dataset. The communication score was calculated as follows:

Communication score=LFC_sort×LFC_whole.

where LFC_sort indicates the log_2_ fold change of the ligand in sorted hepatocytes and LFC_whole indicates the log_2_ fold change of the corresponding receptor in whole liver tissue.

### 4.12 Visium HD Spatial Transcriptomics

Visium HD spatial transcriptomics was performed on FFPE liver sections from no‐treatment control and HTVi‐YAP (Day 3) mice. Samples were processed in parallel. Control sample data were previously reported by Kosaka et al. (2026). Sample preparation was performed according to the manufacturer's protocol (10× Genomics, Pleasanton, California, USA; CG000684) using FFPE liver sections with a DV200 value > 90%. Sections (5 μm) were stained with anti‐ATP1A1 antibody (1:500; ab76020; Abcam, Cambridge, UK) and DAPI, and fluorescence images were acquired using a BZ‐X700 microscope (Keyence). The same stained sections were subsequently transferred to Visium HD slides using the CytAssist instrument (10× Genomics) and used for library preparation. Libraries were sequenced on an AVITI platform (Element Biosciences, San Diego, California, USA).

### 4.13 Visium HD Data Processing and Analysis

Raw sequencing data were processed using Space Ranger (10× Genomics) and converted to Zarr format using spatialdata‐io (v0.1.dev815+g70f5060). Individual cells were segmented as regions of interest (ROIs) using Cellpose (v3.1.1.1) based on ATP1A1 immunofluorescence and DAPI signals. ROI geometries were integrated into a SpatialData object (v0.3.1.dev17+gc2136b3), and gene expression was aggregated within each ROI to quantify transcript abundance per cell. Individual sample datasets were concatenated into a single AnnData object, excluding cells with fewer than 50 detected genes and genes detected in fewer than 100 cells. Batch effects were corrected using scVI (v1.3.0) with a variational autoencoder architecture consisting of two hidden layers and 10 latent dimensions, using a negative binomial gene likelihood. A neighborhood graph (*k* = 5) was constructed from the batch‐corrected latent space and used for Leiden clustering (resolution = 0.5) and UMAP visualization (minimum distance = 0.1). Cell types were annotated manually based on canonical marker gene expression. Major cell populations included hepatocytes, liver sinusoidal endothelial cells, Kupffer cells, lymphocytes, cholangiocytes, and stellate cells/fibroblasts. Among HTVi‐YAP‐specific hepatocytes, cells with high expression of YAP–TEAD target genes were defined as YAP_active_hepatocytes, whereas the remaining cells were designated as atypical hepatocytes.

### 4.14 CellChat Analysis

Intercellular communication analysis was performed using the CellChat R package (v1.6.1). To reduce computational burden, data were downsampled to a maximum of 2000 cells per cell type. Ligand–receptor pairs shown in Figure 3b were used for analysis. Communication probabilities were calculated using the truncatedMean method with spatial distance constraints (scale factor = 0.5). Communication networks were visualized as circle plots based on aggregated cell identities.

### 4.15 ATAC‐Seq

For direct comparison between AAV‐YAP and HTVi‐YAP livers, hepatocytes were isolated from each group by collagenase perfusion as described above. For analysis of chromatin accessibility in YAP‐active hepatocytes, Rosa26‐tdTomato mice were subjected to HTVi with pLive‐Empty‐IRES‐Cre (HTVi‐empty) or pLive‐YAP(2SA)‐IRES‐Cre (HTVi‐YAP). Hepatocytes were isolated by collagenase perfusion, and tdTomato‐positive hepatocytes were purified by FACS. ATAC‐seq library preparation, sequencing, and primary data processing were performed by Takara Bio Inc. (Shiga, Japan) and Active Motif Inc. (Carlsbad, California, USA). Libraries were sequenced on an Illumina NextSeq 500 platform with paired‐end 42 bp reads. Reads were aligned to the reference genome using BWA (v0.7.12), and accessible chromatin regions were identified using MACS2 (v2.1.0). Overlapping peaks across samples were merged into “Merged Regions” and annotated to nearby genes based on RefSeq gene tables from the NCBI database. Regions located within 10 kb upstream or downstream of a gene were assigned to that gene. Differential accessibility analysis was performed using DESeq2, and regions with FDR < 0.1 were considered significant. Motif enrichment analysis was performed using HOMER on the top 2500 differentially accessible regions ranked by shrunken log_2_ fold change.

### 4.16 Cell Culture

HEK293T and HepG2 cells were maintained in high‐glucose Dulbecco's modified Eagle medium (DMEM; Nacalai Tesque, Kyoto, Japan) supplemented with 10% fetal bovine serum (FBS; Cell Culture Bioscience) and 1% penicillin–streptomycin. Cells were cultured at 37°C in a humidified incubator with 5% CO_2_.

### 4.17 In Situ Proximity Ligation Assay

HepG2 cells were transfected with pCMV‐YAP(2SA). In situ proximity ligation assay (PLA) was performed using Duolink In Situ Detection Reagents (Sigma‐Aldrich, St. Louis, Missouri, USA) according to the manufacturer's instructions. Cells were permeabilized with 0.5% Triton X‐100 in PBS for 30 min at RT and blocked with Duolink Blocking Solution for 60 min at 37°C. Samples were incubated with rabbit anti‐CTCF antibody (1:200; Cell Signaling Technology, Danvers, Massachusetts, USA) and mouse anti‐YAP antibody (H9; 1:400; sc‐271134; Santa Cruz Biotechnology, Dallas, Texas, USA) for 120 min at 37°C. PLA probes (Anti‐Rabbit PLUS and Anti‐Mouse MINUS) were then applied for 60 min at 37°C, followed by ligation for 30 min and amplification for 100 min at 37°C. Samples were mounted with Duolink In Situ Mounting Medium with DAPI and imaged using an FV4000 confocal microscope (Evident, Tokyo, Japan).

### 4.18 Co‐Immunoprecipitation

HEK293T cells transfected with pCMV‐FLAG‐Myc‐TEAD4, pCMV‐Myc‐YAP(2SA), and pCMV‐Myc‐CTCF were washed with PBS and lysed in TNE lysis buffer. Lysates were incubated on ice for 10 min and clarified by centrifugation at 15,000 rpm for 10 min at 4°C. A portion of the supernatant was collected as input and mixed with 4× sample buffer. For immunoprecipitation, the remaining lysate was incubated with anti‐FLAG antibody (1 μL; F1804; Sigma‐Aldrich) for 3 h at 4°C with rotation, followed by incubation with Dynabeads Protein G (Invitrogen, Thermo Fisher Scientific, Waltham, Massachusetts, USA) overnight at 4°C. Beads were washed five times with TNE wash buffer containing 1.0% NP‐40 and boiled in 1× sample buffer at 95°C for 5 min to elute bound proteins.

### 4.19 Western Blotting

Input and immunoprecipitated samples were separated by SDS‐PAGE and transferred to membranes. Signals were detected using SuperSignal West Pico Chemiluminescent Substrate (Thermo Fisher Scientific) and visualized with a ChemiDoc MP imaging system (Bio‐Rad Laboratories, Hercules, California, USA). Uncropped images of the immunoblots are shown in Supplementary Figure 6.

### 4.20 Structural Prediction

Predicted structures of trimeric complexes consisting of CTCF, YAP, and TEAD family members (TEAD1–4) were generated using the AlphaFold Server (Google DeepMind; https://alphafoldserver.com/). Full‐length human protein sequences were obtained from UniProt. Predicted protein–protein interactions and model confidence were evaluated based on Predicted Aligned Error (PAE) plots.

### 4.21 Statistical Analysis

Statistical analyses were performed using GraphPad Prism 9 (GraphPad Software, San Diego, California, USA). Comparisons between two groups were performed using unpaired two‐tailed Student's *t*‐test. Comparisons among three or more groups were performed using one‐way analysis of variance (ANOVA). A *p* value < 0.05 was considered statistically significant.

## Author Contributions

**Daiki Hatakeyama:** conceptualization, investigation, formal analysis, writing – original draft, writing – review and editing. **Yoshimi Okamoto‐Uchida:** conceptualization, formal analysis, supervision, writing – review and editing, writing – original draft, visualization, project administration, validation, investigation, funding acquisition. **Misaki Kosaka:** investigation, formal analysis, writing – review and editing. **Haruka Hirose:** formal analysis, writing – review and editing. **Satoshi Kofuji:** investigation, writing – review and editing. **Keiko Danzaki:** investigation, writing – review and editing. **Yoshiki Endo:** investigation, writing – review and editing. **Michiko Nakagawa:** investigation, writing – review and editing. **Akinori Kanai:** investigation, writing – review and editing, formal analysis. **Yutaka Suzuki:** investigation, resources, writing – review and editing. **Takeshi Yokoo:** formal analysis, writing – review and editing. **Shuji Terai:** formal analysis, writing – review and editing. **Teppei Shimamura:** resources, formal analysis, writing – review and editing. **Hiroshi Nishina:** project administration, conceptualization, funding acquisition, writing – original draft, writing – review and editing, supervision.

## Funding

This work was supported by the JSPS KAKENHI Grant Numbers [JP23K06628 (Y.O.), JP22H04925 (PAGS) (Y.O.), JP26K10125 (Y.O.), and JP24K02177 (H.N.)]; AMED (JP22‐24fk0310508) (H.N.); Priority Research Areas in Medical and Dental Sciences, Science Tokyo (Y.O.); Young Innovative Medical Scientist Unit, Science Tokyo (Y.O.); Pharmacodynamics Research Foundation (Y.O.); SECOM Science and Technology Foundation (H.N.); Medical Research Center Initiative for High Depth Omics, Science Tokyo (H.N.); Nanken‐Kyoten, Science Tokyo (H.N.); and Multilayered Stress Diseases (JPMXP1323015483), Science Tokyo (H.N.).

## Disclosure

All authors have reviewed and approved the final version of the manuscript and agree to its submission to *Genes to Cells*.

## Ethics Statement

All animal experiments were approved by the Institutional Animal Care and Use Committee of the Institute of Science Tokyo (Approval No. A2025‐019A; approval date: April 1, 2025) and were performed in accordance with institutional guidelines.

## Conflicts of Interest

The authors declare no conflicts of interest.

## Supporting information


**Supplementary Table 1.** RNA‐seq data (No treatment, HTVi‐Empty, HTVi‐YAP, AAV‐YAP).
**Supplementary Table 2**. Primer sequences used for RT‐qPCR.

**Supplementary Figure 1.** Schematic overview of the study questions. Upper: Ectopic expression of activated YAP in hepatocytes via AAV induces hepatocyte proliferation. Lower: Ectopic expression of activated YAP by HTVi in the context of liver injury leads to hepatocyte elimination. Three key questions remain unresolved: Q1, which genes are induced in YAP‐activated hepatocytes under injury conditions; Q2, how YAP‐activated hepatocytes communicate with macrophages during elimination; and Q3, what mechanisms drive the induction of genes associated with hepatocyte elimination.
**Supplementary Figure 2**. Distinct fates of YAP (2SA)‐expressing hepatocytes following AAV‐ or HTVi‐mediated gene delivery. (a) Immunofluorescence images of mouse liver sections at 3 and 7 days after delivery of activated YAP (2SA) by adeno‐associated virus (AAV) or hydrodynamic tail vein injection (HTVi). Exogenous YAP (2SA) was detected using an anti‐Myc antibody (red), and nuclei were counterstained with DAPI (blue). Scale bar, 100 μm. (b) Quantification of the proportion of Myc‐tagged YAP (2SA)‐positive hepatocytes among total hepatocytes at 3 and 7 days after YAP (2SA) delivery. Data are presented as mean ± SD (*n* = 3). (c) Serum levels of alanine aminotransferase (ALT), aspartate aminotransferase (AST), and lactate dehydrogenase (LDH) at 0, 6, and 24 h after YAP (2SA) delivery by AAV or HTVi. Data are presented as mean ± SD (*n* = 3).
**Supplementary Figure 3**. Functional annotation of genes enriched in pathways related to cell cycle control, RHO GTPase signaling, and immunoregulatory cell–cell interactions. (a) Cell cycle checkpoint regulators. Cdkn2c inhibits the G1/S transition by preventing the association of Cdk4/6 with Cyclin D. In response to DNA double‐strand breaks (DSBs), H2AX is phosphorylated to γH2AX, which, together with BRCA1, contributes to the activation of the S phase checkpoint. Gadd45b suppresses the G2/M transition through inhibition of Cyclin B–Cdk1 activity. (b) Regulators of RHO GTPase signaling and actin dynamics. Rab3b is implicated in the regulation of RHO GTPase signaling. Arhgap11a, acting as a Rho GTPase‐activating protein (GAP), promotes inactivation of RHO GTPases and is associated with actin depolymerization. In contrast, Fgd3 functions as a Rho guanine nucleotide exchange factor (GEF), facilitating activation of RHO GTPases and promoting actin polymerization. (c) Genes potentially involved in immunoregulatory interactions between hepatocytes and macrophages. Icam1 mediates adhesion to immune cells. H2‐M3 presents N‐formylated peptides and may contribute to immune recognition of injured cells. Csf1 is involved in macrophage proliferation, survival, and recruitment, and may facilitate macrophage‐mediated elimination of YAP‐active hepatocytes.
**Supplementary Figure 4**. Matrix plot of YAP/TEAD target gene expression across hepatocyte populations. A matrix plot showing the expression of YAP/TEAD downstream target genes in hepatocytes (Hepatocyte 01–06), injured hepatocytes (Atypical hepatocytes), and YAP‐active hepatocytes. Yellow indicates higher expression of the corresponding genes.
**Supplementary Figure 5**. AlphaFold3‐predicted interactions between TEAD4 and COUP‐TFII or CEBP. Predicted aligned error (PAE) maps for TEAD4 in complex with COUP‐TFII or CEBP generated by AlphaFold3. Lower values indicate higher confidence in the predicted interactions.
**Supplementary Figure 6**. Uncropped western blot images corresponding to Figure 5d. Full‐length membranes from the co‐immunoprecipitation assay shown in Figure 5d. Red dashed boxes indicate the regions displayed in the main figure. Lanes 1, 9, 10, and 11: molecular weight markers; Lanes 2–8: samples as indicated.

## Data Availability

The RNA‐seq and ATAC‐seq data generated in this study have been deposited in the DDBJ Sequence Read Archive (DRA) under the BioProject accession number PRJDB42056. The Visium HD data have been deposited in the Genomic Expression Archive (GEA) under the accession number E‐GEAD‐1095. The Control dataset was also used in our previous study (Kosaka et al. 2026).
