# Supplementary material for: A YAP–TEAD–CTCF Transcriptional Regulatory Axis Associated With the Elimination of Injured Hepatocytes From the Liver
Source: Genes Cells. 2026 Oct 4;31(6):e70150. doi: 10.1111/gtc.70150 (PMC13635467; doi:10.1111/gtc.70150)
Supplement: Supplementary file 1 [file GTC-31-0-s002.pdf]

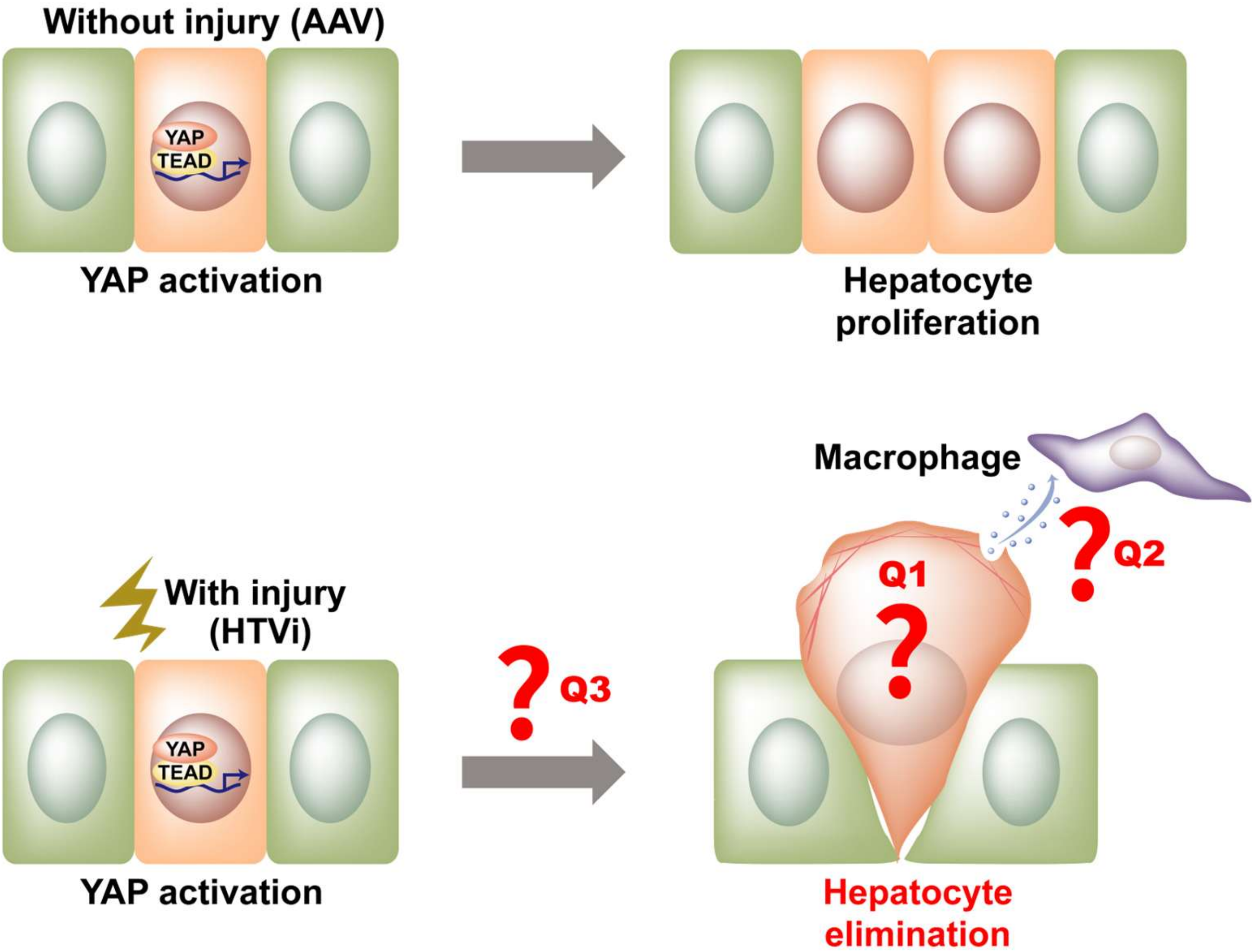

Supplementary Fig. 1 | Schematic overview of the study questions.
Upper: Ectopic expression of activated YAP in hepatocytes via AAV induces hepatocyte proliferation. Lower: Ectopic expression of activated YAP by HTVi in the context of liver injury leads to hepatocyte elimination. Three key questions remain unresolved: Q1, which genes are induced in YAP-activated hepatocytes under injury conditions; Q2, how YAP-activated hepatocytes communicate with macrophages during elimination; and Q3, what mechanisms drive the induction of genes associated with hepatocyte elimination.

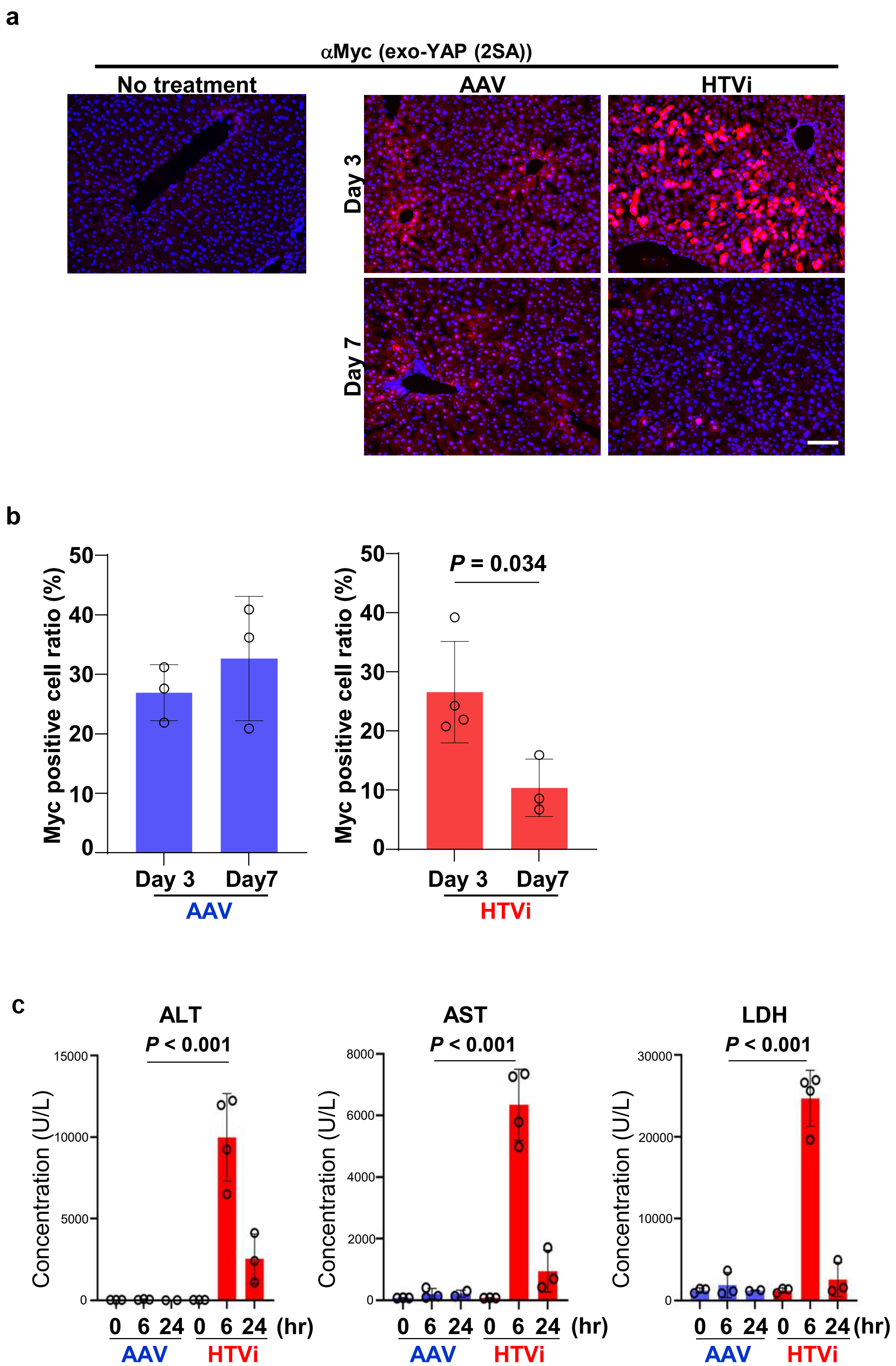

Supplementary Fig. 2 | Distinct fates of YAP (2SA)-expressing hepatocytes following AAV- or HTVi-mediated gene delivery.

(a) Immunofluorescence images of mouse liver sections at 3 and 7 days after delivery of activated YAP (2SA) by adeno-associated virus (AAV) or hydrodynamic tail vein injection (HTVi). Exogenous YAP (2SA) was detected using an anti-Myc antibody (red), and nuclei were counterstained with DAPI (blue). Scale bar, 100 μm. **(b)** Quantification of the proportion of Myc-tagged YAP (2SA)-positive hepatocytes among total hepatocytes at 3 and 7 days after YAP (2SA) delivery. Data are presented as mean ± SD (n = 3). **(c)** Serum levels of alanine aminotransferase (ALT), aspartate aminotransferase (AST), and lactate dehydrogenase (LDH) at 0, 6, and 24 h after YAP (2SA) delivery by AAV or HTVi. Data are presented as mean ± SD (n = 3).

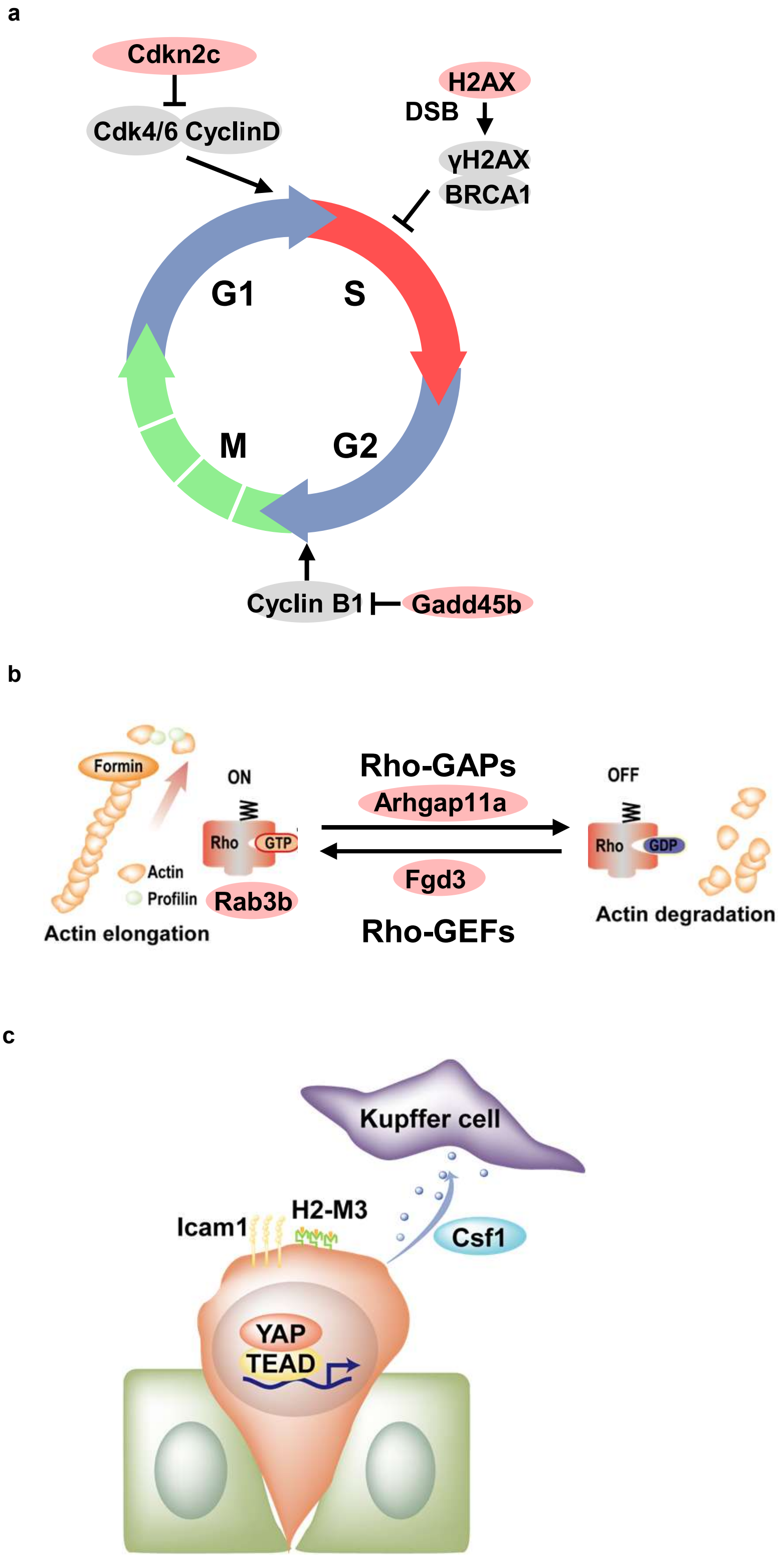

Supplementary Fig. 3 | Functional annotation of genes enriched in pathways related to cell cycle control, RHO GTPase signaling, and immunoregulatory cell–cell interactions.

(a) Cell cycle checkpoint regulators. Cdkn2c inhibits the G1/S transition by preventing the association of Cdk4/6 with Cyclin D. In response to DNA double-strand breaks (DSBs), H2AX is phosphorylated to γH2AX, which, together with BRCA1, contributes to the activation of the S phase checkpoint. Gadd45b suppresses the G2/M transition through inhibition of Cyclin B–Cdk1 activity. **(b)** Regulators of RHO GTPase signaling and actin dynamics. Rab3b is implicated in the regulation of RHO GTPase signaling. Arhgap11a, acting as a Rho GTPase-activating protein (GAP), promotes inactivation of RHO GTPases and is associated with actin depolymerization. In contrast, Fgd3 functions as a Rho guanine nucleotide exchange factor (GEF), facilitating activation of RHO GTPases and promoting actin polymerization**.** **(c)** Genes potentially involved in immunoregulatory interactions between hepatocytes and macrophages. Icam1 mediates adhesion to immune cells. H2-M3 presents N-formylated peptides and may contribute to immune recognition of injured cells. Csf1 is involved in macrophage proliferation, survival, and recruitment, and may facilitate macrophage-mediated elimination of YAP-active hepatocytes.

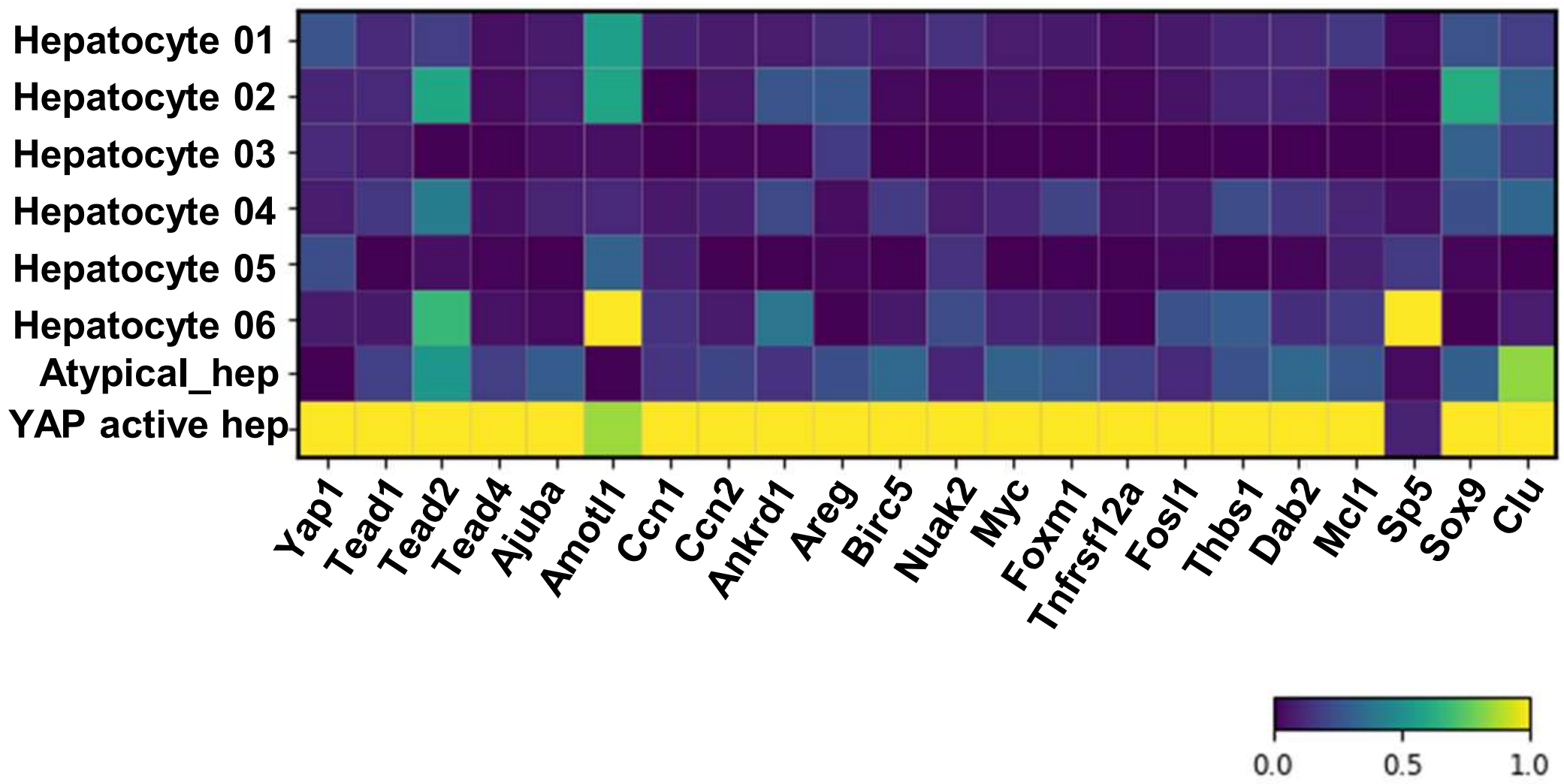

Supplementary Fig. 4 | Matrix plot of YAP/TEAD target gene expression across hepatocyte populations.

A matrix plot showing the expression of YAP/TEAD downstream target genes in hepatocytes (Hepatocyte 01–06), injured hepatocytes (Atypical hepatocytes), and YAP-active hepatocytes. Yellow indicates higher expression of the corresponding genes.

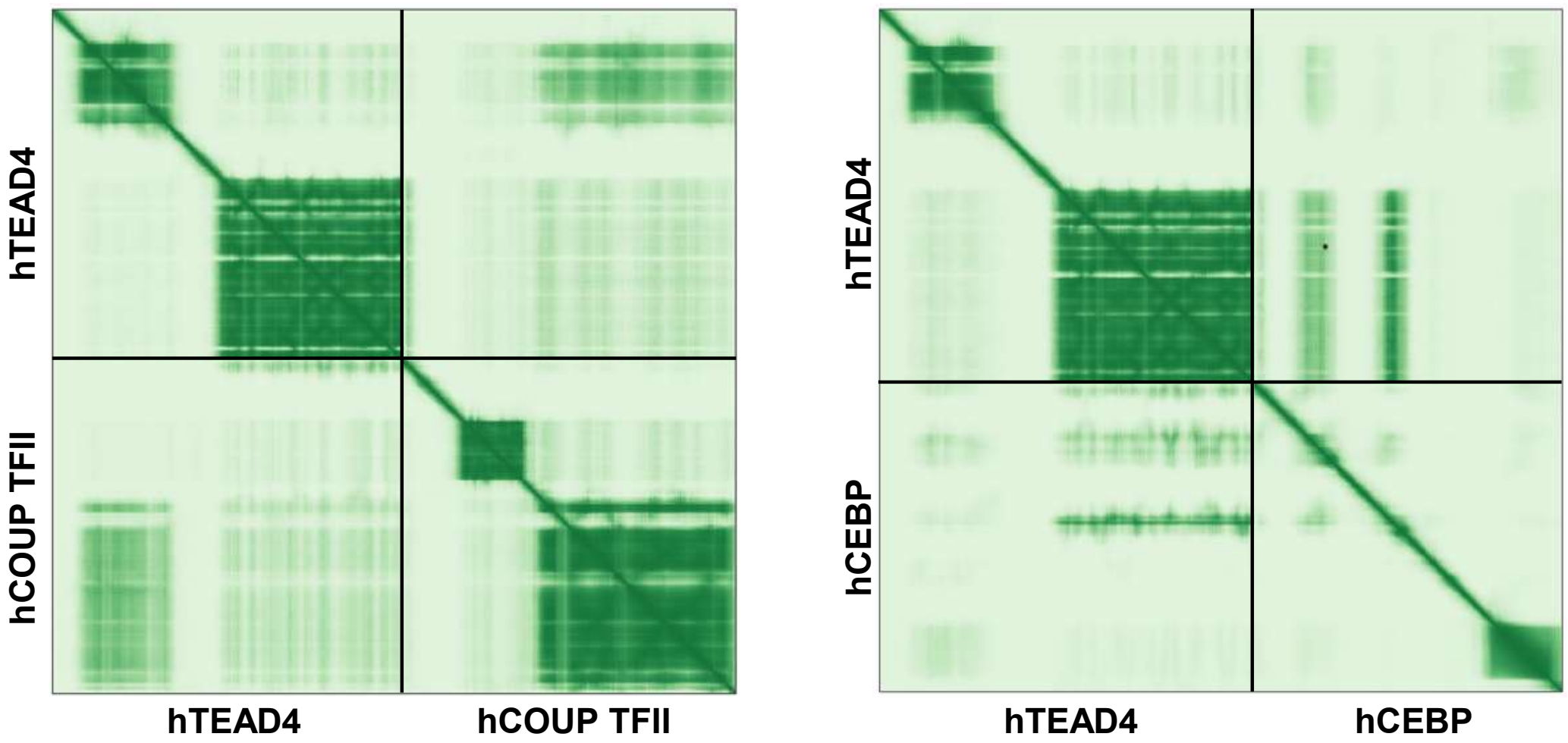

Supplementary Fig. 5 | AlphaFold3-predicted interactions between TEAD4 and COUP-TFII or CEBP.

Predicted aligned error (PAE) maps for TEAD4 in complex with COUP-TFII or CEBP generated by AlphaFold3. Lower values indicate higher confidence in the predicted interactions.

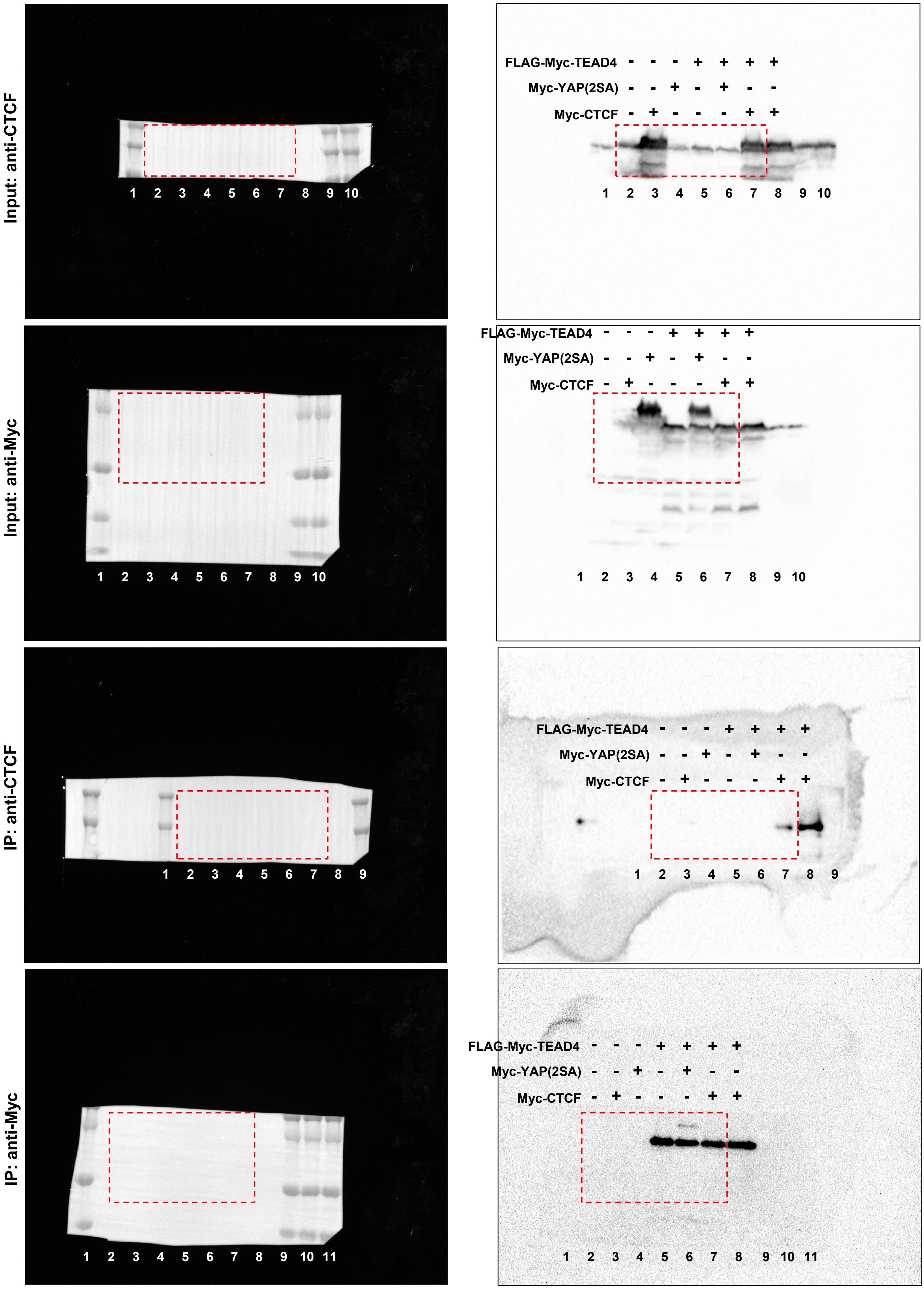

Supplementary Fig. 6 | Uncropped western blot images corresponding to Fig. 5d.
Full-length membranes from the co-immunoprecipitation assay shown in Fig. 5d. Red dashed boxes indicate the regions displayed in the main figure. Lanes 1, 9, 10 and 11: molecular weight markers; lanes 2–8: samples as indicated.
